# Broadband NIR photostimulated luminescence nanoprobes based on CaS:Eu^2+^,Sm^3+^ nanocrystals†
†Electronic supplementary information (ESI) available: Fig. S1–S22. See DOI: 10.1039/c9sc01321k


**DOI:** 10.1039/c9sc01321k

**Published:** 2019-05-10

**Authors:** Yu Gao, Renfu Li, Wei Zheng, Xiaoying Shang, Jiaojiao Wei, Meiran Zhang, Jin Xu, Wenwu You, Zhuo Chen, Xueyuan Chen

**Affiliations:** a CAS Key Laboratory of Design and Assembly of Functional Nanostructures , Fujian Key Laboratory of Nanomaterials , Fujian Institute of Research on the Structure of Matter , Chinese Academy of Sciences , Fuzhou , Fujian 350002 , China . Email: zhengwei@fjirsm.ac.cn ; Email: xchen@fjirsm.ac.cn ; Fax: +86 591 63179421 ; Tel: +86 591 63179421; b School of Physical Science and Technology , ShanghaiTech University , Shanghai 201210 , China; c University of Chinese Academy of Sciences , Beijing 100049 , China; d State Key Laboratory of High Performance Ceramic and Superfine Microstructures , Shanghai Institute of Ceramics , Chinese Academy of Sciences , Shanghai 200050 , China

## Abstract

Near-infrared (NIR) photostimulated luminescence (PSL) nanocrystals (NCs) have recently evoked considerable interest in the field of biomedicine, but are currently limited by the controlled synthesis of efficient PSL NCs. Herein, we report for the first time the controlled synthesis of CaS:Eu^2+^,Sm^3+^ NIR PSL NCs through a high-temperature co-precipitation method. The role of Sm^3+^ co-doping and the effect of thermal annealing on the optical properties of the NCs as well as the charging and discharging processes, the trap depth distribution, and the underlying PSL mechanism are comprehensively surveyed by means of photoluminescence, persistent luminescence, thermoluminescence, and PSL spectroscopies. The as-prepared NCs exhibit intense PSL of Eu^2+^ at 650 nm with a fast response to stimulation in a broad NIR region from 800 nm to 1600 nm, a duration time longer than 2 h, and an extremely low power density threshold down to 10 mW cm^–2^ at 980 nm. Furthermore, by taking advantage of the intense NIR PSL, we demonstrate the application of CaS:Eu^2+^,Sm^3+^ NCs as sensitive luminescent nanoprobes for biotin receptor-targeted cancer cell imaging. These results reveal the great promise of CaS:Eu^2+^,Sm^3+^ nanoprobes for autofluorescence-free bioimaging, and also lay the foundation for future design of efficient NIR PSL nanoprobes towards versatile bioapplications.

## Abstract 2

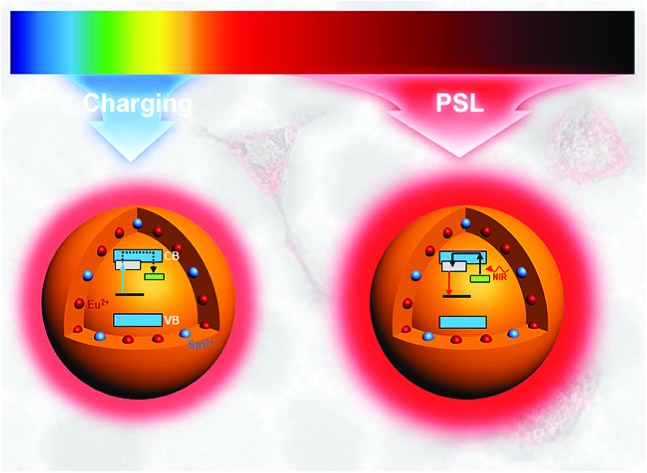
A broadband NIR photostimulated luminescence nanoprobe with an ultralow power density threshold is developed based on CaS:Eu^2+^,Sm^3+^ nanocrystals.

## 1. Introduction

Photostimulated luminescence (PSL) phosphors can store excitation energy and release it as light emission upon infrared light irradiation.^1^–^5^ The physical origin of PSL is similar to that of persistent luminescence (PersL) except that the traps of PSL materials are deeper than those of their PersL analogs, which cannot be thermally activated at room temperature.^6^–^9^ Over the past decades, numerous efforts have been devoted to the design of efficient PSL phosphors for applications in radiation dosimeters, optical storage, infrared sensors, and luminescent paints.^10^–^14^ Recently, nanoscale PSL materials have attracted particular attention in the field of biomedicine.^15^–^20^ These PSL nanocrystals (NCs) are able to emit light on demand by using near-infrared (NIR) light with lower energy such as a 980 nm diode laser for stimulation.^21^–^25^ The feature of anti-Stokes photon upconversion of PSL NCs, along with the high tissue penetration depth and the absence of autofluorescence in biological specimens under NIR stimulation, makes them extremely suitable for use as sensitive luminescent nanoprobes for various bioapplications.^26^–^30^

Rare earth (RE) doped alkaline-earth sulfides (AES), as famous electron-trapping materials, have shown great promise in many technological fields because of their excellent cathodoluminescence, electroluminescence, photoluminescence (PL), PersL, and PSL properties.^31^–^33^ Specifically, RE-doped CaS NCs such as CaS:Eu^2+^,Dy^3+^ and CaS:Eu^2+^,Sm^3+^,Mn^2^ are regarded as promising PSL nanoprobes for *in vitro* biodetection and *in vivo* bioimaging owing to their superior PSL characteristics.^28^,^29^ However, currently the controlled synthesis of highly efficient RE-doped CaS PSL NCs with a broad NIR response range and ultralow stimulation power density threshold remains notoriously difficult. A systematic investigation of RE-doped CaS PSL NCs from the controlled synthesis, fundamental PSL photophysics to bioapplications, which is of vital importance for their optical performance optimization and for future design of efficient PSL nanoprobes, remains unexplored so far.

Herein, we develop a novel strategy for the controlled synthesis of CaS:Eu^2+^ and CaS:Eu^2+^,Sm^3+^ NCs through a high-temperature co-precipitation method. The role of Sm^3+^ co-doping and the effect of thermal annealing on the PL, PersL, and PSL of the NCs are investigated. The charging and discharging processes and the trap depth distribution, as well as the underlying mechanism for the PSL of the NCs are comprehensively surveyed through thermoluminescence (TL), PersL and PSL spectroscopies. Furthermore, by conjugating with a layer of biotinylated phospholipids, we demonstrate the application of CaS:Eu^2+^,Sm^3+^ NCs as sensitive NIR PSL nanoprobes for biotin receptor-targeted cancer cell imaging, thus revealing the great potential of these NIR PSL nanoprobes for autofluorescence-free bioimaging.

## 2. Experimental

### 2.1. Chemicals and materials

Ca(CH_3_COO)_2_·H_2_O (≥99%), Eu(CH_3_COO)_3_·4H_2_O (99.99%), and Sm(CH_3_COO)_3_·4H_2_O (99.99%) were purchased from Aladdin (China). Oleic acid (OA), oleylamine (OAm), trioctylamine (TOA), and *N*,*N*′-diphenylthiourea (DPTU) were purchased from Sigma-Aldrich (China). Cyclohexane, chloroform, acetone, and ethanol were purchased from Sinopharm Chemical Reagent Co., China. 1,2-Distearoyl-*sn*-glycero-3-phosphoethanol-amine-*N*-[biotin(polyethyleneglycol)-2000] (DSPE-PEG-biotin) phospholipids were purchased from Shanghai Yare Biotech Inc., China. All chemical reagents were of analytical grade and used as received without further purification unless otherwise noted. Distilled water was used throughout the experiments.

### 2.2. Synthesis of CaS:Eu^2+^ and CaS:Eu^2+^,Sm^3+^ NCs

CaS:Eu^2+^ and CaS:Eu^2+^,Sm^3+^ NCs were synthesized through a unique high-temperature co-precipitation method. In a typical synthesis of CaS:0.06%Eu^2+^, 0.006%Sm^3+^ NCs, 1 mmol Ca(CH_3_COO)_2_·H_2_O and 60 μL aqueous solution of Eu(CH_3_COO)_3_·4H_2_O (0.01 M) and Sm(CH_3_COO)_3_·4H_2_O (0.001 M) were mixed with 2 mL of OA, 12 mL of OAm, and 6 mL of TOA in a 50 mL three-necked round-bottom flask. The resulting mixture was heated to 120 °C under a N_2_ flow with constant stirring for 30 min to remove the residual water and oxygen, and then heated to 160 °C and stirred for 30 min to form a clear solution. After cooling down to room temperature (RT), 10 mL of ethanol solution containing 3 mmol of DPTU was added and the solution was stirred at 70 °C for 30 min to remove ethanol. After ethanol was evaporated, the resulting solution was heated to 320 °C under a N_2_ flow with vigorous stirring for 60 min, and then cooled down to RT. The obtained NCs were precipitated by addition of 20 mL of ethanol, collected by centrifugation, washed with ethanol several times, and finally dried at 60 °C for 24 h in a vacuum.

### 2.3. Postsynthetic treatment of CaS:Eu^2+^ and CaS:Eu^2+^,Sm^3+^ NCs

To improve their optical performance, the as-synthesized CaS:Eu^2+^ and CaS:Eu^2+^,Sm^3+^ NCs were annealed at 850 °C for 1 h in a furnace under a H_2_/N_2_ (5%/95%) reducing atmosphere. After annealing, the NCs were thoroughly ground, dispersed in ethanol, and ultrasonicated for 20 min. Thereafter, the suspension was centrifuged at 5000 rpm for 3 min to precipitate the large and aggregated NCs. The supernatant was collected and dried at 50 °C to evaporate ethanol. The yield of the NCs after centrifugation was determined to be higher than 50 wt% of their original NCs before centrifugation. The obtained ligand-free NCs were finally re-dispersed in 10 mL of ethanol.

### 2.4. Synthesis of biotinylated CaS:Eu^2+^ and CaS:Eu^2+^,Sm^3+^ NCs

In a typical synthesis, 10 mg of ligand-free CaS:Eu^2+^,Sm^3+^ NCs were mixed with 30 mg of DSPE-PEG-biotin in 5 mL of chloroform. The mixture solution was allowed for vigorous stirring for 8 h, and then centrifuged at 12 000 rpm for 5 min. The biotinylated NCs were purified by washing with chloroform several times, and finally dispersed in distilled water for subsequent application.

### 2.5. PSL bioimaging of cancer cells based on CaS:Eu^2+^,Sm^3+^ NCs

HeLa cell and L-02 cell lines were purchased from the Shanghai Institute of Cell Biology, Chinese Academy of Sciences, and were routinely maintained in RPMI-1640 (GIBCO BRL), supplemented with 10% (v/v) heat-inactivated fetal calf serum, penicillin (100 U mL^–1^) and streptomycin (100 U mL^–1^) at 37 °C under humidified air containing 5% CO_2_. HeLa and L-02 cells were respectively seeded into culture plates and allowed to adhere for 24 h. After washing several times with PBS (Phosphate Buffered Saline, pH 7.2), the cells were incubated in culture medium (RPMI-1640) containing 0.5 mg mL^–1^ of biotinylated CaS:Eu^2+^,Sm^3+^ NCs at 37 °C for 2 h under 5% CO_2_, and then washed with PBS sufficiently to remove excess NCs. Cell imaging was performed on a confocal laser scanning microscope (Nikon Ti-E&C2). The cells were illuminated with a household white LED (1 W) for 5 min to charge the NCs and then excited with a 980 nm diode laser, and the PSL signal was collected in the red channel (640–680 nm).

### 2.6. Cytotoxicity assay of biotinylated CaS:Eu^2+^,Sm^3+^ NCs

Human normal liver (L-02) cells were seeded into a 96-well culture plate at 2 × 10^4^ cells per well and cultured at 37 °C in humidified air containing 5% CO_2_ for 24 h before addition of different concentrations of biotinylated CaS:Eu^2+^,Sm^3+^ NCs (32, 63, 125, 250, and 500 μg mL^–1^ in RPMI 1640). The cells were then incubated at 37 °C under 5% CO_2_ for another 24 h. One control column in the plate was filled with fresh culture medium only. The cells were then washed twice with sterile PBS before addition of fresh medium. Methylthiazolyltetrazolium (MTT) was subsequently applied to the cells followed by incubation at 37 °C under 5% CO_2_ for 4 h. The OD490 value of each well was measured on a multimodal microplate reader (Synergy 4, BioTek). The following formula was applied to calculate the inhibition rate of cell growth: cell viability (%) = (mean of the absorbance value of the treatment group/mean of absorbance value of control) × 100%.

### 2.7. Characterization

Powder X-ray diffraction (XRD) patterns of the samples were collected on an X-ray diffractometer (MiniFlex 2, Rigaku) with Cu Kα1 radiation (*λ* = 0.154 nm). Both the low- and high-resolution transmission electron microscopy (TEM) measurements were performed by using a TECNAI G^2^ F20 TEM equipped with an energy dispersive X-ray (EDX) spectrometer. The chemical composition of the samples was analyzed by inductively coupled plasma-atomic emission spectroscopy (ICP-AES, Ultima2, Jobin Yvon). The zeta potential and hydrodynamic diameter of the NCs were determined by dynamic light scattering (DLS) measurement (Nano ZS ZEN3600, Malvern). Fourier transform infrared spectra (FTIR) were recorded in KBr discs on a Magna 750 FTIR spectrometer. For optical characterization of the NCs, PL excitation spectra, PL emission spectra, PL decays, PersL emission spectra, PersL decays, TL glow curves, PSL emission spectra, and PSL decays were acquired on an FLS980 spectrometer (Edinburgh) equipped with both continuous xenon (450 W) and pulsed flash lamps. PL, PersL, and PSL photographs of the NCs were taken by using a Canon 70D digital camera without using any filter. The absolute PL quantum yields (QYs) of the NCs were measured by employing a standard barium sulfate coated integrating sphere (150 mm in diameter, Edinburgh) as the sample chamber that was mounted on the FLS920 spectrometer (Edinburgh) with the entry and output port of the sphere located in 90° geometry from each other in the plane of the spectrometer. A standard tungsten lamp was used to correct the optical response of the instrument. All the spectral data were recorded at RT unless otherwise noted, and corrected for the response of both the spectrometer and the integrating sphere.

For PLQYs, PersL emission spectra, PersL decays, PSL emission spectra, and PSL decays, the samples were irradiated by using a household white LED (1 W) for 5 min before the measurements. For TL measurement, the samples were placed on the thermal stage and then exposed to continuous xenon lamp irradiation at 470 nm for 5 min at 300 K, and immediately after the cease of excitation, the temperature was allowed to fall to 100 K and then rise to 650 K with a heating rate of 1 K s^–1^. The Eu^2+^ emission at 650 nm was monitored. The TL glow curves were obtained by transforming the time base of the curves to a temperature base utilizing the linear heating rate employed in the measurement. PSL stimulation spectra were measured with a customized UV to mid-infrared steady-state and phosphorescence lifetime spectrometer (FSP920-C, Edinburgh) equipped with a digital oscilloscope (TDS3052B, Tektronix) and a tunable mid-band Optical Parametric Oscillator (OPO) pulsed laser as the excitation source (410–2400 nm, 10 Hz, pulse width ≤ 5 ns, Vibrant 355II, OPOTEK).

## 3. Results and discussion

### 3.1. Structure and morphology

**Fig. 1:**
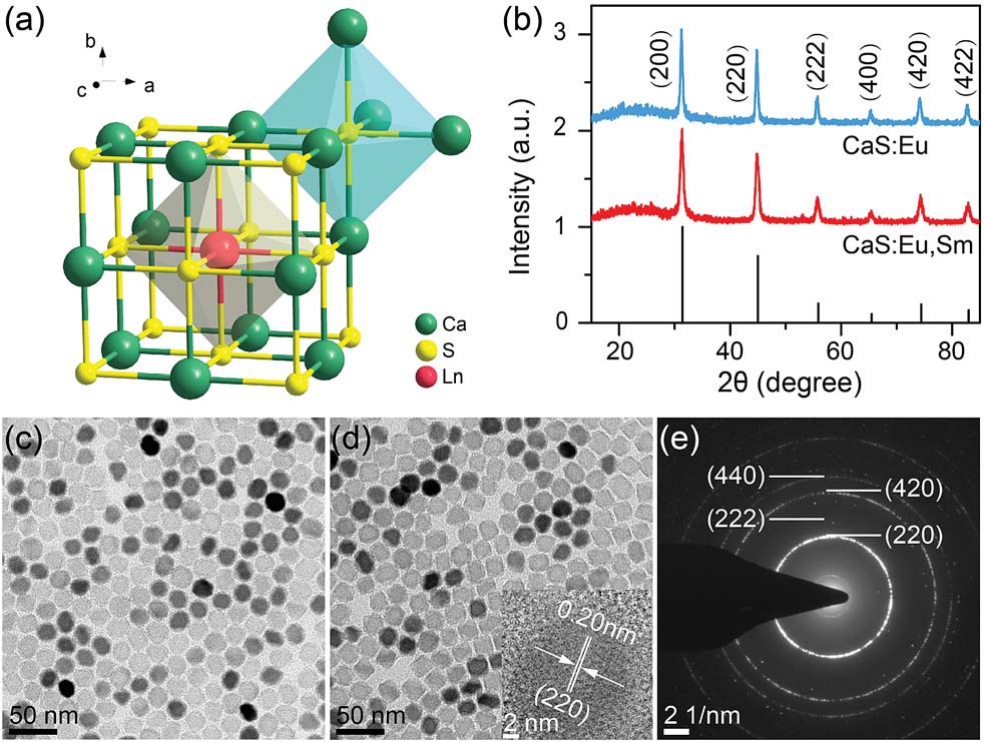
(a) Arrangement of calcium and sulphur atoms in the CaS structure and the crystallographic site for Ln dopants. (b) Powder XRD patterns of CaS:0.06%Eu^2+^ and CaS:0.06%Eu^2+^, 0.006%Sm^3+^ NCs. The bottom lines represent the standard XRD pattern of cubic CaS (JCPDS no. 008-0464). TEM images of (c) CaS:0.06%Eu^2+^ and (d) CaS:0.06%Eu^2+^, 0.006%Sm^3+^ NCs. The inset in (d) shows the corresponding HRTEM image. (e) SAED pattern of CaS:0.06%Eu^2+^, 0.006%Sm^3+^ NCs.

The CaS crystal has a rock-salt structure (space group *Fm*3[combining macron]*m*) with Ca^2+^ ions surrounded by six S^2–^ ions that form the edges of an octahedron (Fig. 1a).^34^ The Eu^2+^ and Sm^3+^ ions occupy the octahedral Ca^2+^ site.^35^ High-quality CaS:Eu^2+^ and CaS:Eu^2+^,Sm^3+^ NCs were synthesized through a novel high-temperature co-precipitation method by employing DPTU as the sulphur source, OA and OAm as the surfactant, and TOA as the solvent. The as-synthesized NCs are hydrophobic and can be readily dispersed in a variety of nonpolar organic solvents such as cyclohexane. Powder XRD patterns (Fig. 1b) show that all diffraction peaks of the NCs can be well indexed to cubic CaS (JCPDS no. 008-0464), with mean sizes of 23.9 ± 0.9 nm and 22.7 ± 1.4 nm for CaS:Eu^2+^ and CaS:Eu^2+^,Sm^3+^ NCs, respectively, according to the Debye–Scherrer equation (Fig. S1†). TEM images (Fig. 1c and d) show that both CaS:Eu^2+^ and CaS:Eu^2+^,Sm^3+^ NCs are cubic with mean sizes of 24.6 ± 1.5 nm and 22.1 ± 1.3 nm, respectively (Fig. S2†), which are very well consistent with the mean sizes determined from their XRD patterns. The high-resolution TEM image (inset of Fig. 1d) exhibits clear lattice fringes with an observed *d* spacing of 0.20 nm, in good agreement with the lattice spacing of the (220) plane of cubic CaS. The SAED pattern of the NCs (Fig. 1e) displays intense diffraction rings of cubic CaS, confirming the pure phase and high crystallinity of the resulting NCs. Compositional analyses through the EDX spectrum and ICP-AES reveal 0.058 mol% of Eu^2+^ in CaS:0.06%Eu^2+^ NCs and 0.059 mol% of Eu^2+^ and 0.006 mol% of Sm^3+^ in CaS:0.06%Eu^2+^, 0.006%Sm^3+^ NCs (Fig. S3†), which are generally consistent with the nominal doping concentrations.

### 3.2. PL properties of the as-synthesized CaS:Eu^2+^ and CaS:Eu^2+^,Sm^3+^ NCs

**Fig. 2:**
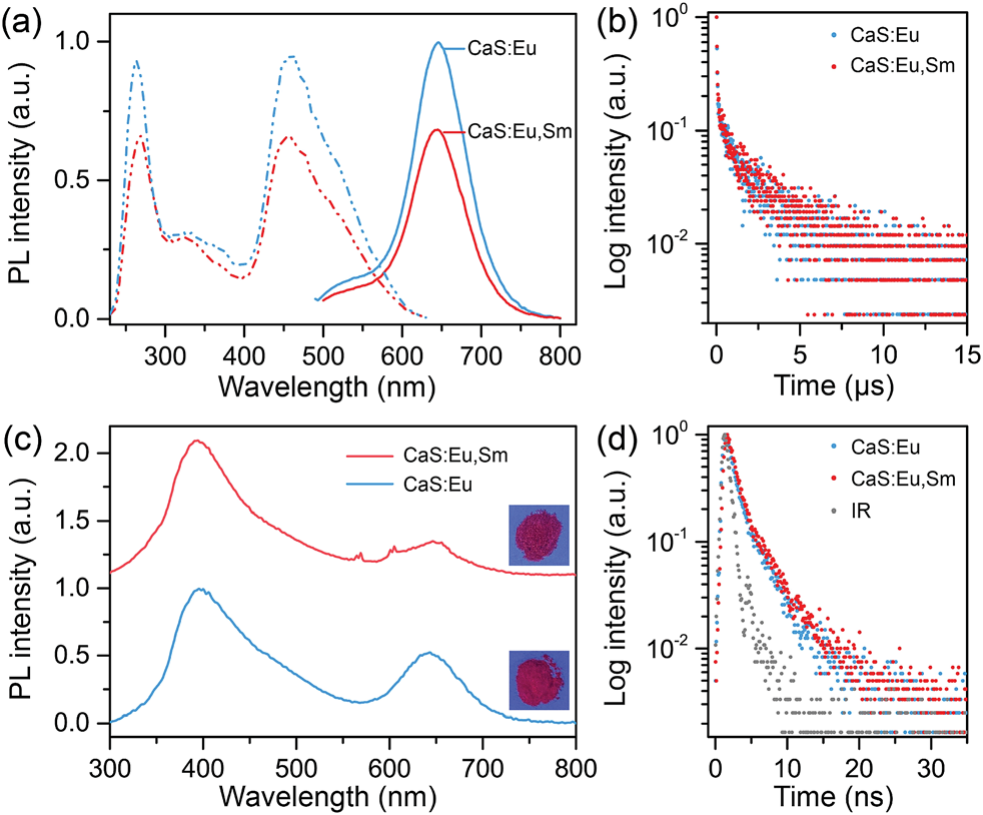
(a) PL excitation spectra (dash-dotted line, *λ*_em_ = 650 nm), PL emission spectra (solid line, *λ*_ex_ = 470 nm), and (b) PL decays (*λ*_em_ = 650 nm) of the as-synthesized CaS:0.06%Eu^2+^ and CaS:0.06%Eu^2+^, 0.006%Sm^3+^ NCs. (c) PL emission spectra (*λ*_ex_ = 270 nm) and (d) PL decays (*λ*_em_ = 400 nm) of the as-synthesized CaS:0.06%Eu^2+^ and CaS:0.06%Eu^2+^, 0.006%Sm^3+^ NCs. The insets in (c) show the corresponding photographs of the NCs under 254 nm UV lamp illumination. IR in (d) represents the instrument response.

Fig. 2a shows the PL excitation and emission spectra of the as-synthesized CaS:Eu^2+^ and CaS:Eu^2+^,Sm^3+^ NCs. Under excitation at 470 nm, a broad emission band centered at 650 nm was observed, corresponding to the electronic transition from the lowest T_2g_ level of the 4f^6^5d^1^ state to the ^8^S_7/2_ level of the 4f^7^ ground state of Eu^2+^ occupying the octahedral site (O_h_) in the CaS lattice.^36^,^37^ The optimal doping concentration of Eu^2+^ was determined to be 0.06 mol% (Fig. S4†). Sm^3+^ co-doping was found to decrease the PL intensity of Eu^2+^ in the NCs (Fig. S5†), preserving 64% of the PL intensity of Eu^2+^ singly-doped NCs after co-doping with 0.006 mol% of Sm^3+^. This can be ascribed to the trapping of excitation energy by deep traps created by Sm^3+^ co-doping.^38^–^40^ PL decay curves show a nearly identical PL lifetime of 0.31 μs for Eu^2+^ in CaS:0.06%Eu^2+^ and CaS:0.06%Eu^2+^, 0.006%Sm^3+^ NCs (Fig. 2b). By monitoring the Eu^2+^ emission at 650 nm, three excitation bands with central wavelengths at 270, 325, and 470 nm were detected. The excitation bands at 470 nm and 270 nm can be assigned to the 4f^7^ → 4f^6^5d^1^ (T_2g_) transition of Eu^2+^ and the host absorption of CaS, respectively, suggesting an efficient energy transfer from the CaS host to Eu^2+^;^41^ while the excitation band at 325 nm is related to the absorption of S^2–^ deficiency related defects formed during the wet-chemical synthesis.^35^

Fig. 2c compares the PL emission spectra of CaS:Eu^2+^ and CaS:Eu^2+^,Sm^3+^ NCs upon above-bandgap excitation at 270 nm. Both the NCs displayed a typical Eu^2+^ emission band at 650 nm and a similar broad emission band centered at 400 nm with an identical PL lifetime of 1.8 ns (Fig. 2d), which coincides well with the emission of the undoped CaS NCs and can be ascribed to the S^2–^ deficiency related defect emission (Fig. S6†). Specifically, a set of sharp emission peaks corresponding to the electronic transitions from ^4^G_5/2_ to ^6^H_5/2_, ^6^H_7/2_, and ^6^H_9/2_ of Sm^3+^ at 568, 605, and 656 nm, respectively, were explicitly observed in CaS:Eu^2+^,Sm^3+^ NCs (Fig. S7†), indicating an efficient energy transfer from the CaS host to Sm^3+^.^42^ It is worth mentioning that, besides the steady-state PL, we also observed weak PersL (or afterglow) from Eu^2+^ with a duration time of about 10 min in CaS:Eu^2+^ and CaS:Eu^2+^,Sm^3+^ NCs after the samples were illuminated with a 254 nm UV lamp or a household white LED for 5 min (Fig. S8†).

### 3.3. Persistent luminescence properties of the annealed CaS:Eu^2+^ and CaS:Eu^2+^,Sm^3+^ NCs

**Fig. 3:**
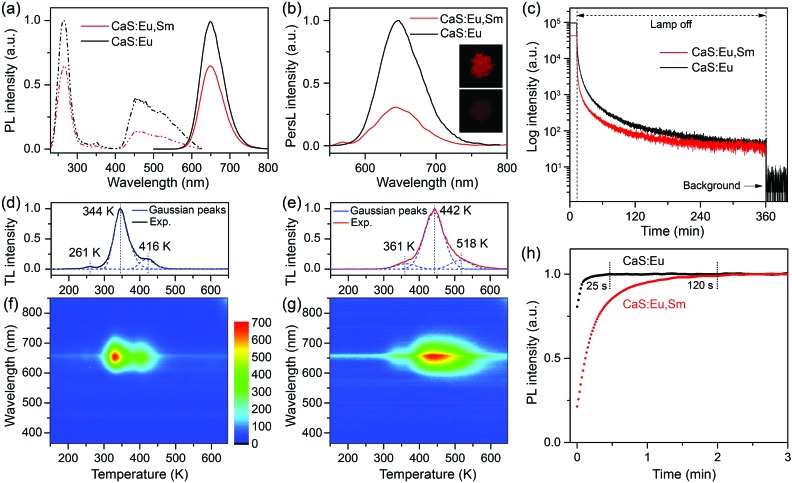
(a) PL excitation spectra (dash-dotted line, *λ*_em_ = 650 nm) and PL emission spectra (solid line, *λ*_ex_ = 470 nm) of the annealed CaS:0.06%Eu^2+^ and CaS:0.06%Eu^2+^, 0.006%Sm^3+^ NCs. (b) PersL emission spectra and (c) PersL decay curves (*λ*_em_ = 650 nm) of the NCs after illumination with a white LED for 5 min. The insets in (b) show the PersL photographs of (top) CaS:0.06%Eu^2+^ and (below) CaS:0.06%Eu^2+^, 0.006%Sm^3+^ NCs recorded at 1 min after the cease of excitation. TL glow curves and the corresponding wavelength-temperature contour plots of (d and f) CaS:0.06%Eu^2+^ and (e and g) CaS:0.06%Eu^2+^, 0.006%Sm^3+^ NCs. (h) PL rise curves (*λ*_ex_ = 470 nm, *λ*_em_ = 650 nm) of CaS:0.06%Eu^2+^ and CaS:0.06%Eu^2+^, 0.006%Sm^3+^ NCs after emptying the traps by heating the samples at 350 °C for 10 min.

To improve the optical properties of CaS:Eu^2+^ and CaS:Eu^2+^,Sm^3+^ NCs, we annealed the NCs at 850 °C for 1 h under a H_2_/N_2_ reducing atmosphere. The annealed NCs preserved the cubic phase and high crystallinity of their pristine NCs, while the monodispersity of the NCs was sacrificed with some particle aggregation. The mean size of the annealed CaS:Eu^2+^,Sm^3+^ NCs was estimated to be 34.6 ± 4.2 nm (Fig. S9†). After annealing, the PL intensities of CaS:Eu^2+^ and CaS:Eu^2+^,Sm^3+^ NCs were remarkably enhanced by factors of 101 and 43, with absolute PLQYs increasing from 0.5% and 0.2% to 45.8% and 15.1%, respectively (Fig. S10†). The drastically enhanced PL of the NCs after annealing can be ascribed to the increased particle size and the removal of surface defects and high-energy vibrational groups which deteriorate seriously the PL of the NCs. Fig. 3a shows the PL excitation and emission spectra of the annealed CaS:Eu^2+^ and CaS:Eu^2+^,Sm^3+^ NCs, which resemble those of their pristine NCs except for the intensified host excitation band at 270 nm and the disappeared defect-related excitation and emission bands at 325 nm and 400 nm, respectively (Fig. S11†). This suggests that thermal annealing can effectively eliminate the detrimental defects in the lattice of the NCs induced by wet-chemical synthesis. Notably, thermal annealing may bring about new lattice defects such as sulphur vacancies in the NCs which can serve as electron traps for the PersL of the NCs (Fig. S12†). As a result, upon above-bandgap excitation at 270 nm, more excitation energy can transfer from the conduction band of CaS to the luminescent center Eu^2+^ ions and the electron traps instead of the detrimental defects, leading to enhanced PL and PersL from the NCs.

Upon illumination with a 254 nm UV lamp or a white LED for 5 min, the annealed CaS:Eu^2+^ and CaS:Eu^2+^,Sm^3+^ NCs displayed intense red PersL (insets of Fig. 3b), in marked contrast to the negligibly weak PersL in their pristine NCs. PersL emission spectra of the NCs recorded at 1 min after the cease of excitation show identical emission patterns of the characteristic Eu^2+^ emission to their steady-state PL emission spectra (Fig. 3b), revealing unambiguously that the PersL of the NCs originates from the 4f^7^ → 4f^6^5d^1^ (T_2g_) transition of Eu^2+^. PersL decay curves of the NCs show a slow afterglow decay over a long-time window beyond 6 h (Fig. 3c), indicative of a long afterglow time of the NCs. Notably, the PersL intensity and afterglow time of CaS:Eu^2+^,Sm^3+^ NCs decreased significantly in comparison with those of CaS:Eu^2+^ NCs, as a result of deep traps caused by Sm^3+^ co-doping.^38^–^40^ It was also found that the PersL intensity of CaS:Eu^2+^,Sm^3+^ NCs depended critically on the wavelength of the illumination light with the maximum at 470 nm (Fig. S13†).

Fig. 3d and e show the TL glow curves of the annealed CaS:Eu^2+^ and CaS:Eu^2+^,Sm^3+^ NCs, respectively. The TL glow peaks of CaS:Eu^2+^,Sm^3+^ NCs shifted to higher temperatures relative to those of CaS:Eu^2+^ NCs, suggesting deeper traps in CaS:Eu^2+^,Sm^3+^ NCs than in CaS:Eu^2+^ NCs. The wavelength–temperature two-dimensional TL mappings of the NCs show a strong TL glow signal in the wavelength region at around 650 nm and the temperature regions corresponding to those of their TL glow peaks (Fig. 3f and g), confirming the exclusive origin of the PersL from the Eu^2+^ emission and the deep traps in CaS:Eu^2+^,Sm^3+^ NCs.^43^,^44^ By Gaussian fitting to the TL glow curves, three TL glow peaks at 261, 344 and 416 K for CaS:Eu^2+^ NCs and three TL glow peaks at 361, 442 and 518 K for CaS:Eu^2+^,Sm^3+^ NCs were de-convoluted, corresponding to different trap depths in the lattice of the NCs.^45^–^47^ Based on Chen's equation,^48^ the trap depths were calculated to be 0.33, 0.63 and 0.92 eV for CaS:Eu^2+^ NCs and 0.47, 0.76 and 1.08 eV for CaS:Eu^2+^,Sm^3+^ NCs (Fig. S14†). The dominant trap depth of 0.63 eV (344 K) in CaS:Eu^2+^ NCs is close to the thermal activation energy (0.6 eV) for efficient PersL at RT, while the dominant trap depth of 0.76 eV (442 K) in CaS:Eu^2+^,Sm^3+^ NCs is high enough to stably store the excitation energy at RT.^49^ It is worth mentioning that the pre-irradiation time has no significant influence on the TL glow peak positions of both CaS:Eu^2+^ and CaS:Eu^2+^,Sm^3+^ NCs (Fig. S15†), but the TL peaks may shift to higher temperatures with increasing the delay time of measurement due to the gradual release of electrons from the shallow traps at RT (Fig. S16†). After emptying the traps by heating the samples at 350 °C for 10 min, we probe the charging process of the traps through measuring the time-dependent PL of the NCs. Upon excitation, part of the excitation energy is stored by the traps until all traps are filled. As a result, the PL intensities of the NCs would rise with time under continuous excitation.^50^ As shown in Fig. 3h, the PL intensities of the NCs with empty traps show a gradual increase with time under continuous excitation at 470 nm, reflecting the charging process of the traps. It was observed that the PL rise time (120 s) of CaS:Eu^2+^,Sm^3+^ NCs was much longer than that (25 s) of CaS:Eu^2+^ NCs due to the slow charging process of the deep traps in CaS:Eu^2+^,Sm^3+^ NCs. Such a long PL rise time of CaS:Eu^2+^,Sm^3+^ NCs reveals the excellent energy storage capability of the NCs.^51^,^52^

### 3.4. PSL properties of the annealed CaS:Eu^2+^,Sm^3+^ NCs

**Fig. 4:**
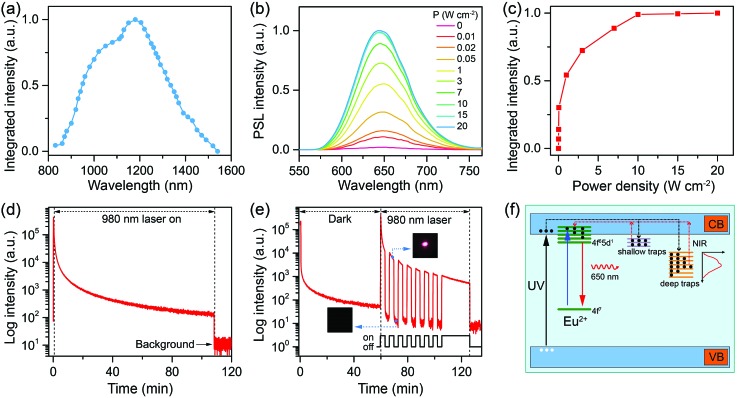
(a) PSL stimulation spectrum of the annealed CaS:0.06%Eu^2+^, 0.006%Sm^3+^ NCs by monitoring the Eu^2+^ emission at 650 nm. (b) PSL emission spectra and (c) integrated PSL intensities of the charged CaS:0.06%Eu^2+^, 0.006%Sm^3+^ NCs upon irradiation with a 980 nm diode laser at different power densities. (d) PSL decay curve (*λ*_em_ = 650 nm) of the charged CaS:0.06%Eu^2+^, 0.006%Sm^3+^ NCs under continuous laser irradiation at 980 nm with a power density of 10 W cm^–2^. (e) PSL decay curve (*λ*_em_ = 650 nm) of the charged CaS:0.06%Eu^2+^, 0.006%Sm^3+^ NCs under periodical laser irradiation at 980 nm with a power density of 10 W cm^–2^ after the NCs underwent 60 min of afterglow decay. The insets show the photographs of the NCs when the laser was on and off, respectively. (f) Schematic illustration of the PSL mechanism in CaS:Eu^2+^,Sm^3+^ NCs. The solid-line arrows denote electronic transitions, and the dashed-line arrows represent the trapping (black) and de-trapping (red) of electrons.

Although the stored excitation energy in deep traps of CaS:Eu^2+^,Sm^3+^ NCs could not be thermally activated at RT, it can be effectively released upon irradiation with NIR light, resulting in intense PSL from the NCs. To probe the NIR response of the PSL, we measure the PSL stimulation (or excitation) spectrum of CaS:Eu^2+^,Sm^3+^ NCs.^40^ For each data point measurement, the sample was first charged with a white LED for 5 min under identical conditions. Then, the PSL decay was recorded by monitoring the Eu^2+^ emission at 650 nm under continuous excitation at a specific wavelength with a tunable OPO pulsed laser (410–2400 nm) (Fig. S17†). The process of charging and stimulation with different wavelengths was repeated until all PSL decay curves were acquired. The final stimulation spectrum was derived by integrating the PSL decay curves at different stimulation wavelengths. As shown in Fig. 4a, the PSL of CaS:Eu^2+^,Sm^3+^ NCs exhibits a response to stimulation in a broad NIR region from 800 nm to 1600 nm with the maximum at ∼1200 nm (1.03 eV), which is very close to the highest trap depth (1.08 eV) of the NCs determined by TL measurement.

Furthermore, we investigated the effect of excitation power density on the PSL of the charged CaS:Eu^2+^,Sm^3+^ NCs by using a 980 nm diode laser for stimulation. As shown in Fig. 4b, the PSL intensity of the NCs increased gradually with increasing the laser power density and saturated at a power density of 10 W cm^–2^ (Fig. 4c). The increased PSL intensity with increasing the laser power density is due to the fact that more trapped electrons can be activated and pumped to the conduction band of CaS followed by the population of 5d levels of Eu^2+^ at higher photon flux.^53^ Meanwhile, the electrons in deeper traps may fill in the empty trap level. Such electron releasing and filling processes may reach a dynamic equilibrium at a specific pumping photon flux, which results in the saturation of the PSL intensity. Specifically, intense PSL from the NCs was explicitly observed upon NIR stimulation at a power density of 10 mW cm^–2^, indicative of an extremely low power density threshold of the PSL NCs. Such a power density threshold for NIR PSL in CaS:Eu^2+^,Sm^3+^ NCs is much lower than that (>500 mW cm^–2^) commonly used for triggering the nonlinear photon upconversion in lanthanide-doped NCs such as NaYF_4_:Yb^3+^,Er^3+^.^54^–^56^ Moreover, it was found that the PSL of the NCs can last for a long time over 2 h under continuous-wave laser irradiation at the saturated power density of 10 W cm^–2^ (Fig. 4d), which is much longer than that of CaS:Eu^2+^,Dy^3+^ NCs (18 min) previously reported.^53^Fig. 4e shows the PSL response of the charged CaS:Eu^2+^,Sm^3+^ NCs to 980 nm laser stimulation after 60 min of afterglow decay. When the laser was turned on, bright red PSL was observed immediately, while once the laser was turned off, the PSL intensity dropped suddenly to a low value (insets of Fig. 4e), revealing a very fast response of the PSL NCs to NIR light.^57^

Based on the above analyses, we propose a physical model to unveil the PSL mechanism in annealed CaS:Eu^2+^,Sm^3+^ NCs (Fig. 4f). The traps are supposed to be electron traps lying below the conduction band minimum (CBM) of CaS NCs with their nature ascribed to the lattice defects intrinsic to CaS such as sulphur vacancies (
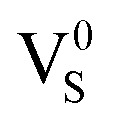
 or 
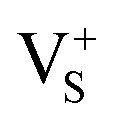
).^58^,^59^ Upon 470 nm (or a white LED) irradiation, the electrons on the 4f levels of Eu^2+^ can be excited to the 5d levels, part of which are slightly below the CBM of CaS.^60^ Then, partial electrons in the 5d levels of Eu^2+^ might return to the 4f levels yielding strong PL emission, and the rest of the excited electrons are trapped by the empty defect levels *via* the relaxation from the CB, resulting in oxidation of Eu^2+^ to Eu^3+^.^61^ Sm^3+^ dopants interact with the defects and lower the defect levels, creating deep traps in the lattice of CaS.^39^ The electrons in the deep traps cannot be released at RT. Instead, by an NIR light stimulation, they can be excited to the CB, and then captured by Eu^3+^ and populated the 5d levels of Eu^2+^, accompanied by light emission through the 4f^7^ → 4f^6^5d^1^ (T_2g_) transition of Eu^2+^.^42^ Under 270 nm (or a 254 nm UV lamp) excitation, the electrons in the valence band (VB) of CaS are pumped to the CB, followed by energy transfer to Eu^2+^ and Sm^3+^ along with PL emission from the ions. Meanwhile, part of the electrons in the CB are trapped by the defect levels, which accounts for the long PSL of the NCs under NIR stimulation.

### 3.5. Targeted cancer cell imaging

To render CaS:Eu^2+^,Sm^3+^ NCs hydrophilic and biocompatible, we coated the NCs with a layer of amphiphilic DSPE-PEG-biotin phospholipids (Lipo). After annealing and thorough grinding, the oleate ligands were removed from the NCs, resulting in ligand-free NCs with positively charged Ca^2+^ ions exposed on the surface. Therefore, DSPE-PEG-biotin Lipo can be conjugated to the NC surface through either electrostatic adsorption or the strong chelation of Ca^2+^ ions.^62^,^63^ The successful conjugation of DSPE-PEG-biotin Lipo to the NC surface was confirmed by FTIR and DLS measurements (Fig. S18 and S19†). The Lipo can not only endow the NCs with water dispersity, but also protect them from degradation in aqueous solution. More importantly, the Lipo-coated NCs preserved the intense PSL of their pristine ligand-free NCs (Fig. S20†).

**Fig. 5:**
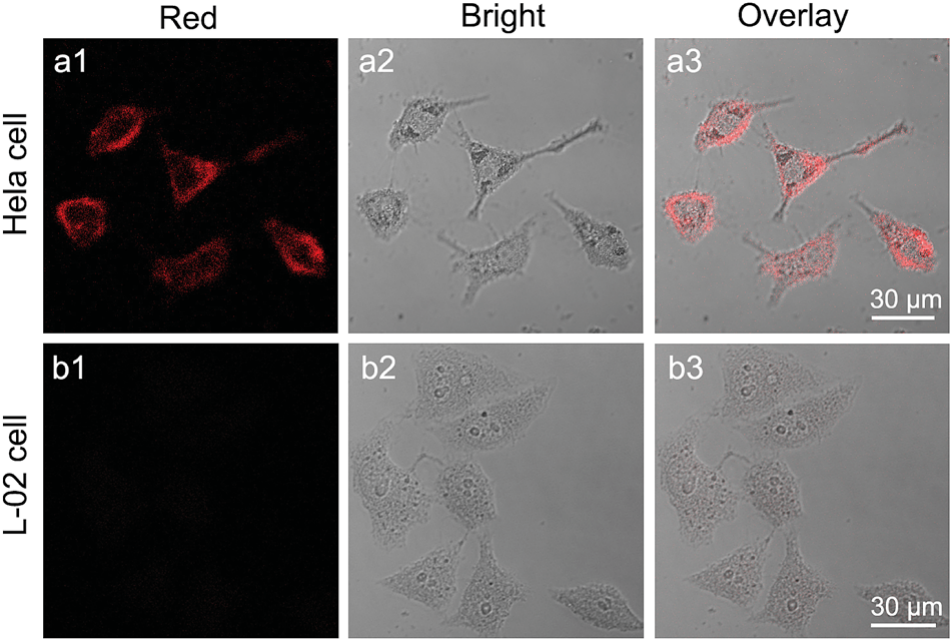
Confocal laser scanning microscopy images of (a1–a3) HeLa cells and (b1–b3) L-02 cells after incubation with Lipo-coated CaS:Eu^2+^,Sm^3+^ NCs (0.5 mg mL^–1^) at 37 °C for 2 h. Intense red PSL of Eu^2+^ (*λ*_em_ = 640–680 nm, *λ*_ex_ = 980 nm) was observed exclusively in HeLa cells after illumination with a white LED for 5 min. Panels 1 and 2 show the red PSL images and bright-field images, respectively. Panel 3 is the overlay images of panels 1 and 2.

By virtue of the specific recognition of biotin with the biotin receptor, these DSPE-PEG-biotin Lipo-coated CaS:Eu^2+^,Sm^3+^ NCs can be exploited as NIR PSL nanoprobes for biotin receptor-targeted cancer cell imaging.^64^ Biotin is a growth promoter at the cellular level, and biotin receptors are over-expressed in a variety of cancer cells, such as colon (Colo-26), lung (M109), and cervical (HeLa) cancer cell lines.^65^ In our experiment, HeLa cells over-expressed with biotin receptors were selected as the target cancer cells and human normal liver (L-02) cells with low-expressed biotin receptors were used as the control. Due to the specific binding between biotin and biotin receptors, the Lipo-coated CaS:Eu^2+^,Sm^3+^ NCs can target HeLa cells. As a result, bright red PSL of Eu^2+^ (red channel) from the NCs can be explicitly observed surrounding HeLa cells under stimulation with a 980 nm diode laser or a 1200 nm ns-pulsed laser (OPO) after the cells were illuminated with a white LED for 5 min (Fig. 5a and S21†). By contrast, the red PSL of the NCs was hardly observed on L-02 cells under otherwise identical conditions due to the lack of specific binding between Lipo-coated NCs and L-02 cells (Fig. 5b). The MTT assay on L-02 cells incubated with Lipo-coated NCs showed a cell viability larger than 98% even at a high NC concentration of 500 μg mL^–1^ (Fig. S22†), indicating that the Lipo-coated CaS:Eu^2+^,Sm^3+^ NCs are biocompatible and nontoxic to live cells. These Lipo-coated CaS:Eu^2+^,Sm^3+^ NCs, featuring a fast PSL response, long PSL duration time (>2 h), and low power density threshold (<10 mW cm^–2^) under NIR stimulation, are ideal for their use as sensitive luminescent nano-bioprobes for autofluorescence-free long-term tracking and *in vivo* bioimaging of various biomolecules.

## 4. Conclusions

In summary, we have systematically investigated CaS:Eu^2+^,Sm^3+^ NIR PSL NCs from the controlled synthesis, fundamental PSL properties to their potential application for autofluorescence-free bioimaging. The CaS:Eu^2+^ and CaS:Eu^2+^,Sm^3+^ NCs, synthesized *via* a novel wet-chemical approach, exhibited weak PL and PersL due to the existence of detrimental lattice and surface defects in the NCs. Thermal annealing was found to be an effective strategy to eliminate these detrimental defects and drastically improve the PL, PerSL, and PSL properties of the NCs. We unraveled that Sm^3+^ co-doping can deepen the trap depth, resulting in remarkably improved PSL of Eu^2+^ at 650 nm with a fast PSL response in a broad NIR region from 800 nm to 1600 nm, a duration time longer than 2 h, and an extremely low power density threshold down to 10 mW cm^–2^ under 980 nm diode laser stimulation. Furthermore, by employing such intense NIR PSL, we exploited the biotinylated CaS:Eu^2+^,Sm^3+^ NCs as sensitive luminescent nanoprobes for biotin receptor-targeted cancer cell imaging, thus revealing the great promise of CaS:Eu^2+^,Sm^3+^ NIR PSL nanoprobes for background-free biosensing. The proposed approach for synthesizing high-performance CaS:Eu^2+^,Sm^3+^ PSL NCs can also be extended to other RE-doped AES nano-systems, which provides a new avenue for the development of highly efficient NIR PSL nanoprobes toward versatile bioapplications.

## Conflicts of interest

There are no conflicts to declare.

## Supplementary Material

Supplementary information
