# Supplementary material for: Broadband NIR photostimulated luminescence nanoprobes based on CaS:Eu2+,Sm3+ nanocrystals
Source: Chem Sci. 2019 May 10;10(21):5452–60. doi: 10.1039/c9sc01321k (PMC6552487; doi:10.1039/c9sc01321k)
Supplement: Supplementary file 1 [file SC-010-C9SC01321K-s001.pdf]

Electronic Supplementary Material (ESI) for Chemical Science.
This journal is © The Royal Society of Chemistry 2019

Broadband NIR photostimulated luminescence nanoprobes based on $CaS:Eu^{2+},Sm^{3+}$ nanocrystals

- Supporting Information

Yu Gao,[a,b,c,d] Renfu Li,[a] Wei Zheng,*[a,c] Jiaojiao Wei,[a] Xiaoying Shang,[a] Meiran Zhang,[a] Jin Xu,[a] Wenwu You,[a] Zhuo Chen,[a,c] and Xueyuan Chen*[a,b,c]

[a]*CAS Key Laboratory of Design and Assembly of Functional Nanostructures, and Fujian Key Laboratory of Nanomaterials, Fujian Institute of Research on the Structure of Matter, Chinese Academy of Sciences, Fuzhou, Fujian 350002, China*

[b]*School of Physical Science and Technology, Shanghai Tech University, Shanghai 201210, China*

[c]*University of Chinese Academy of Sciences, Beijing 100049, China*

[d]*State Key Laboratory of High Performance Ceramic and Superfine Microstructures, Shanghai Institute of Ceramics, Chinese Academy of Sciences, Shanghai 200050, China*

Fax: +86-591-63179421; Tel: +86-591-63179421; E-mail: zhengwei@fjirsm.ac.cn or xchen@fjirsm.ac.cn

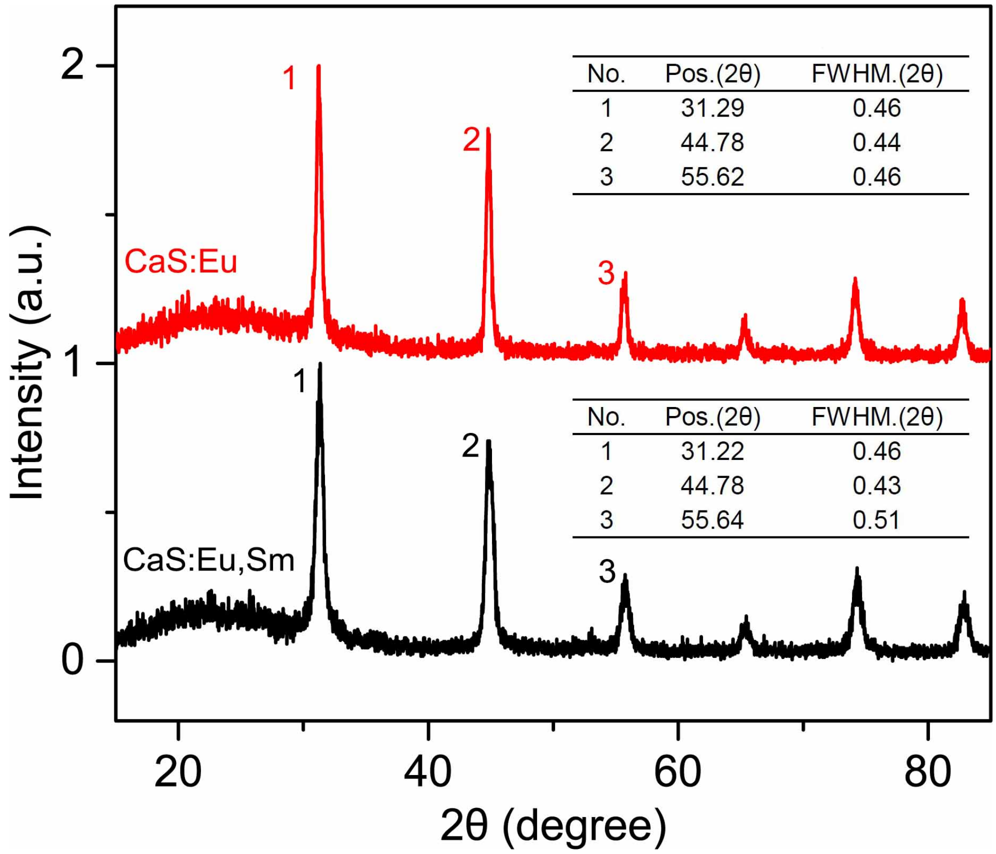

Figure S1. Powder XRD patterns of CaS:0.06%Eu^{2+} and CaS:0.06%Eu^{2+}, 0.006%Sm^{3+} NCs. The insets show the peak position and full width at half maximum (FWHM) of the three major XRD peaks of the NCs. The size of the NCs can be calculated by Debye-Scherrer equation: $d = 0.89\lambda/\beta\cos\theta$, where λ = 0.154 nm and β is the FWHM (in rad) of the XRD peak at the diffraction angle θ. Based on Debye-Scherrer equation, the mean sizes of CaS:0.06%Eu^{2+} and CaS:0.06%Eu^{2+}, 0.006%Sm^{3+} NCs were calculated to be 23.9 ± 0.9 nm and 22.7 ± 1.4 nm, respectively.

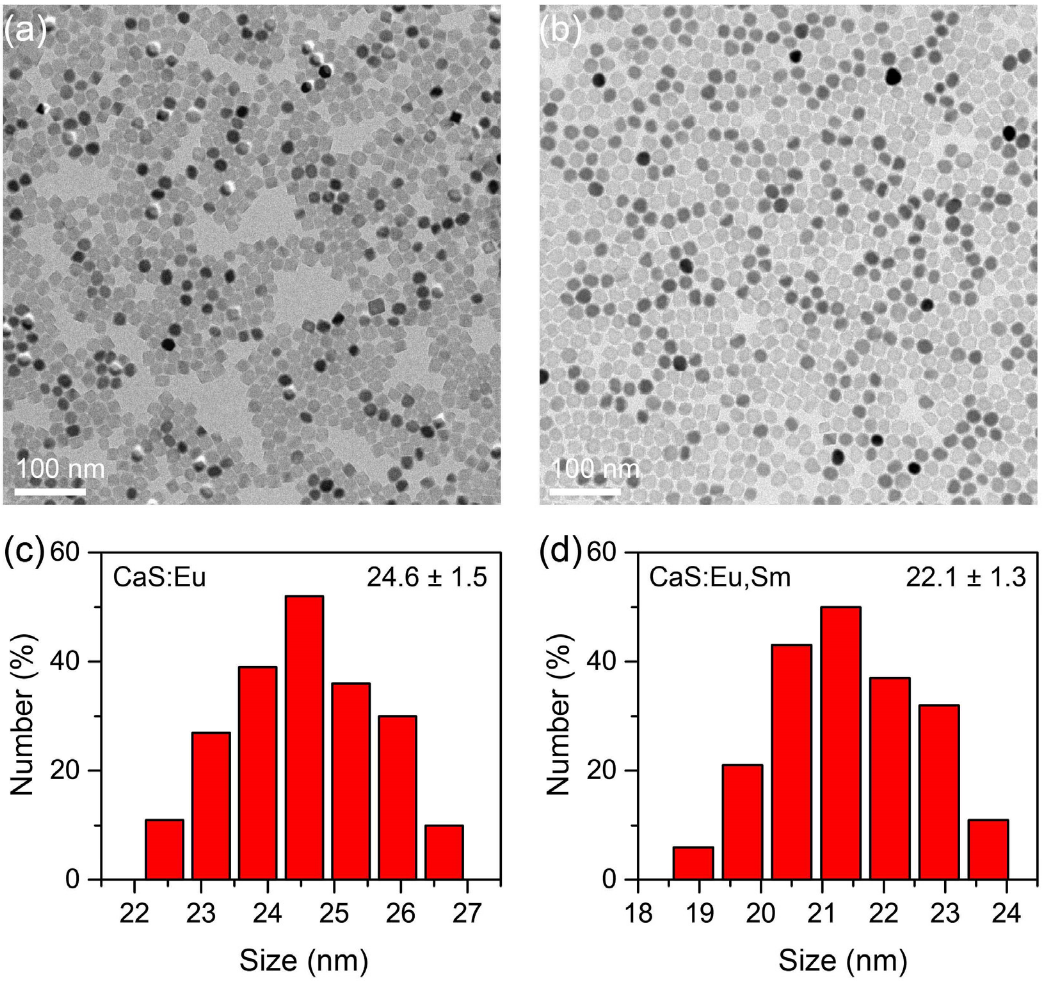

Figure S2. TEM images (~1 mg mL^{-1}) and size distributions of (a, c) CaS:0.06%Eu^{2+} and (b, d) CaS:0.06%Eu^{2+}, 0.006%Sm^{3+} NCs. The size distributions were obtained by randomly calculating 200 particles in the TEM images.

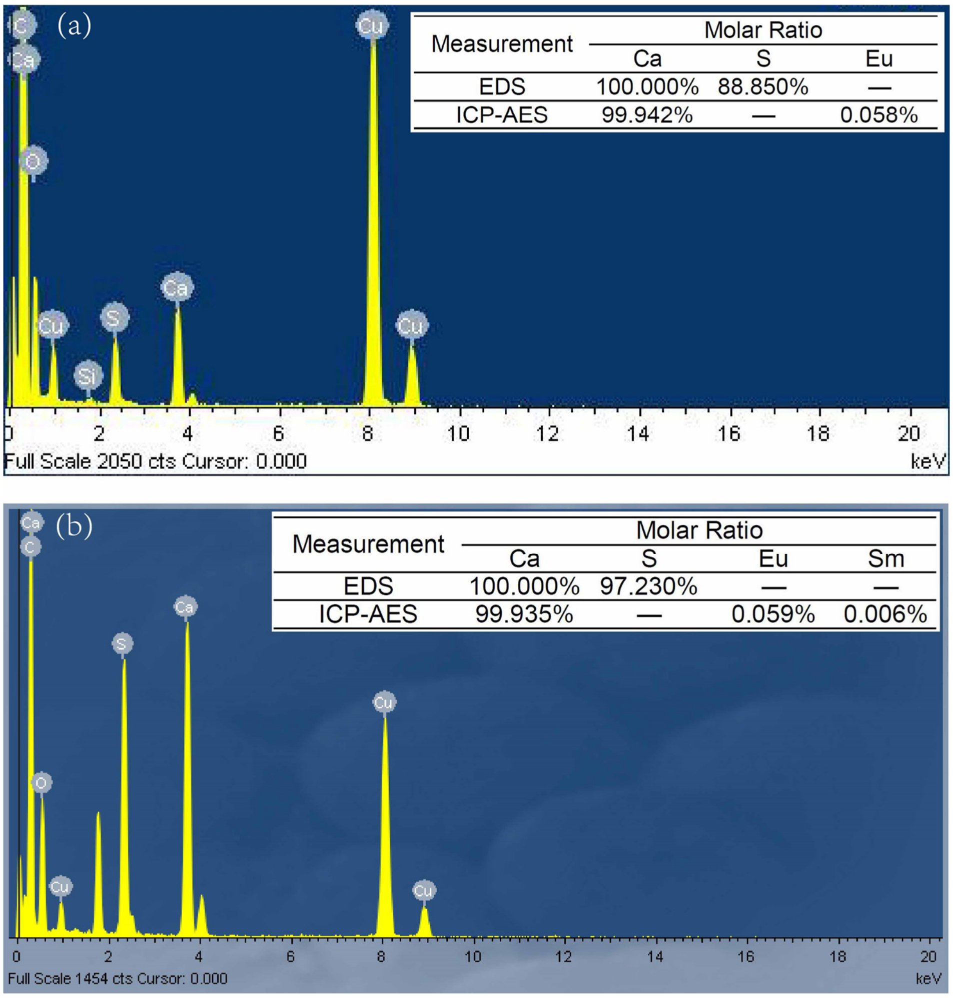

(a)

Measurement	Molar Ratio		
	Ca	S	Eu
EDS	100.000%	88.850%	—
ICP-AES	99.942%	—	0.058%

(b)

Measurement	Molar Ratio			
	Ca	S	Eu	Sm
EDS	100.000%	97.230%	—	—
ICP-AES	99.935%	—	0.059%	0.006%

Figure S3. Energy dispersive X-ray (EDX) spectra of CaS:0.06%Eu^{2+} and CaS:0.06%Eu^{2+},0.006%Sm^{3+} NCs, showing the elements of Ca and S in the NCs. The absence of Eu and Sm dopants is because of their low doping levels that cannot be detected by EDX spectra. The insets show the chemical compositions of the NCs determined by EDX spectra and inductively coupled plasma-atomic emission spectroscopy (ICP-AES), revealing 0.058 mol% of Eu^{2+} in CaS:0.06%Eu^{2+} NCs and 0.059 mol% of Eu^{2+} and 0.006 mol% of Sm^{3+} in CaS:0.06%Eu^{2+}, 0.006%Sm^{3+} NCs.

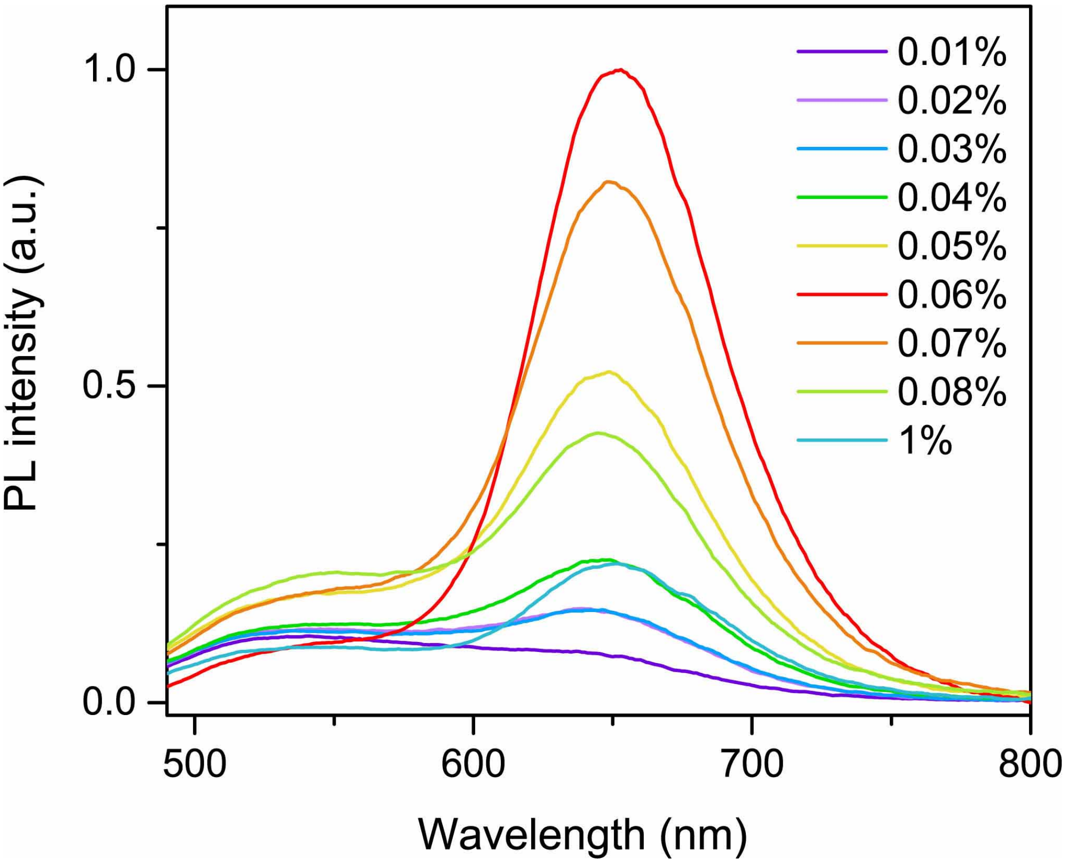

Figure S4. PL emission spectra of CaS:x%Eu^{2+} NCs with different Eu^{2+} concentrations under excitation at 470 nm, from which the optimal doping concentration of Eu^{2+} was determined to be 0.06 mol%.

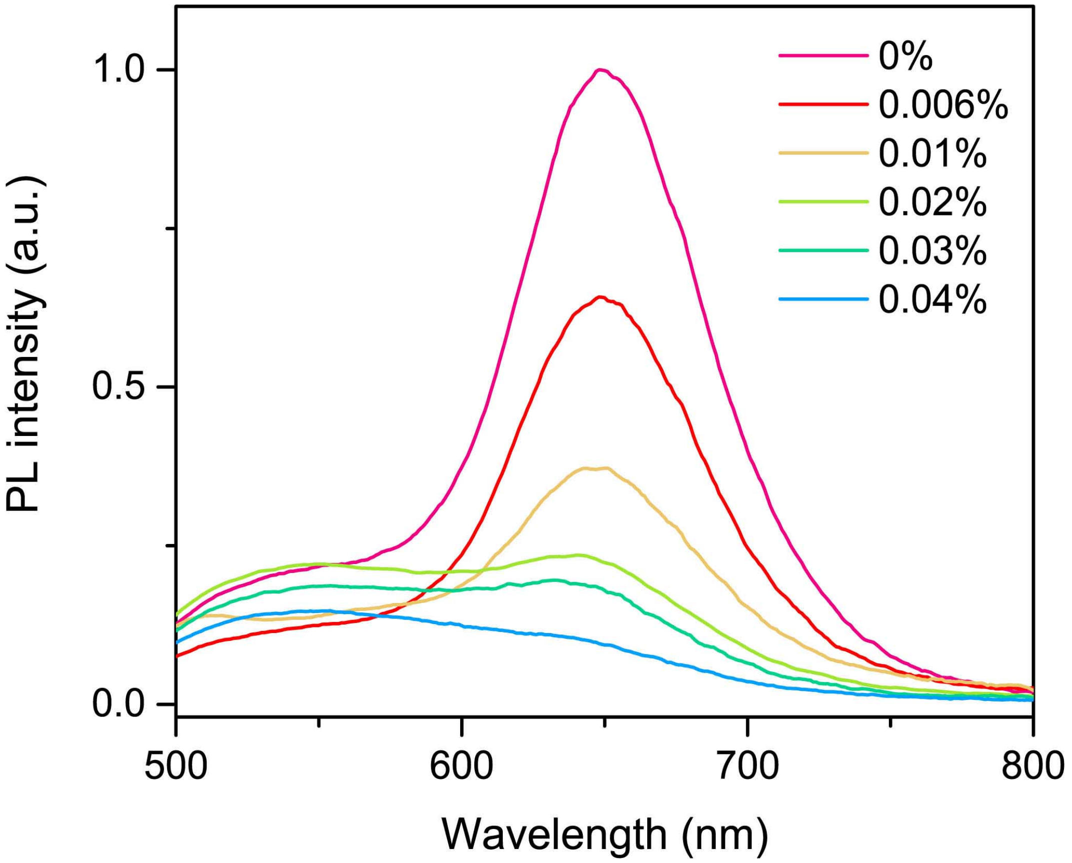

Figure S5. PL emission spectra of CaS:0.06%Eu^{2+}, *y*%Sm^{3+} NCs with different Sm^{3+} concentrations under excitation at 470 nm. The PL intensity of Eu^{2+} decreases gradually with increasing the Sm^{3+} concentration.

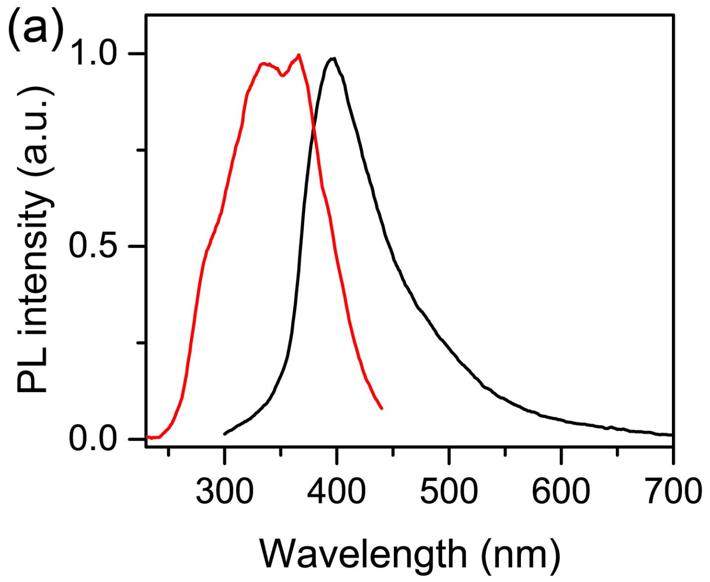

Figure S6. (a) PL excitation spectra (red, λ_{em} = 400 nm), PL emission spectra (black, λ_{ex} = 270 nm), and (b) PL decay of the undoped CaS NCs. IR represents the instrument response. A broad emission band at 400 nm with a PL lifetime of 1.8 ns was detected in the undoped CaS NCs, which can be ascribed to the emission of S^{2-} deficiency related defects formed during the wet-chemical synthesis.

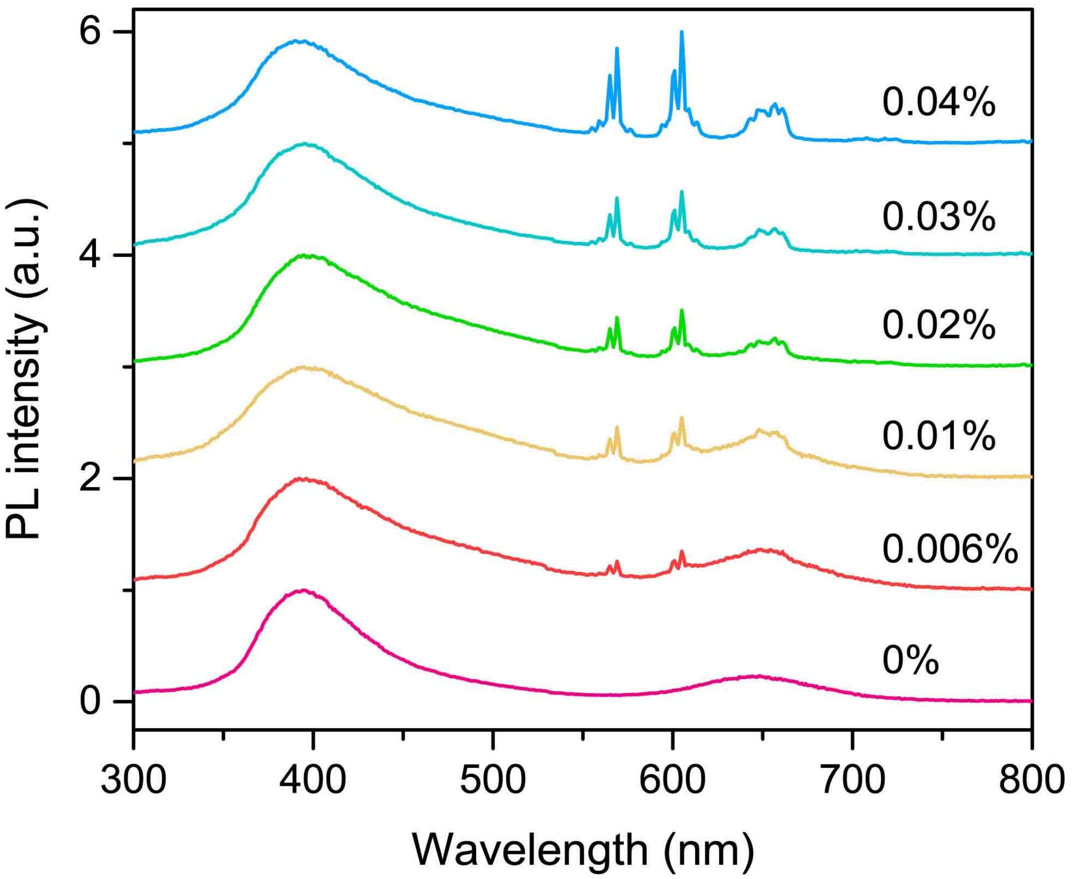

Figure S7. PL emission spectra of CaS:0.06%Eu^{2+}, *y*%Sm^{3+} NCs with different Sm^{3+} concentrations upon above-bandgap excitation at 270 nm. The intensities of the emission peaks corresponding to the electronic transitions from $^4G_{5/2}$ to $^6H_{5/2}$, $^6H_{7/2}$, and $^6H_{9/2}$ of Sm^{3+} at 568, 605, and 656 nm, respectively, increase gradually with increasing the Sm^{3+} concentration, suggesting an efficient energy transfer from CaS host to Sm^{3+}.

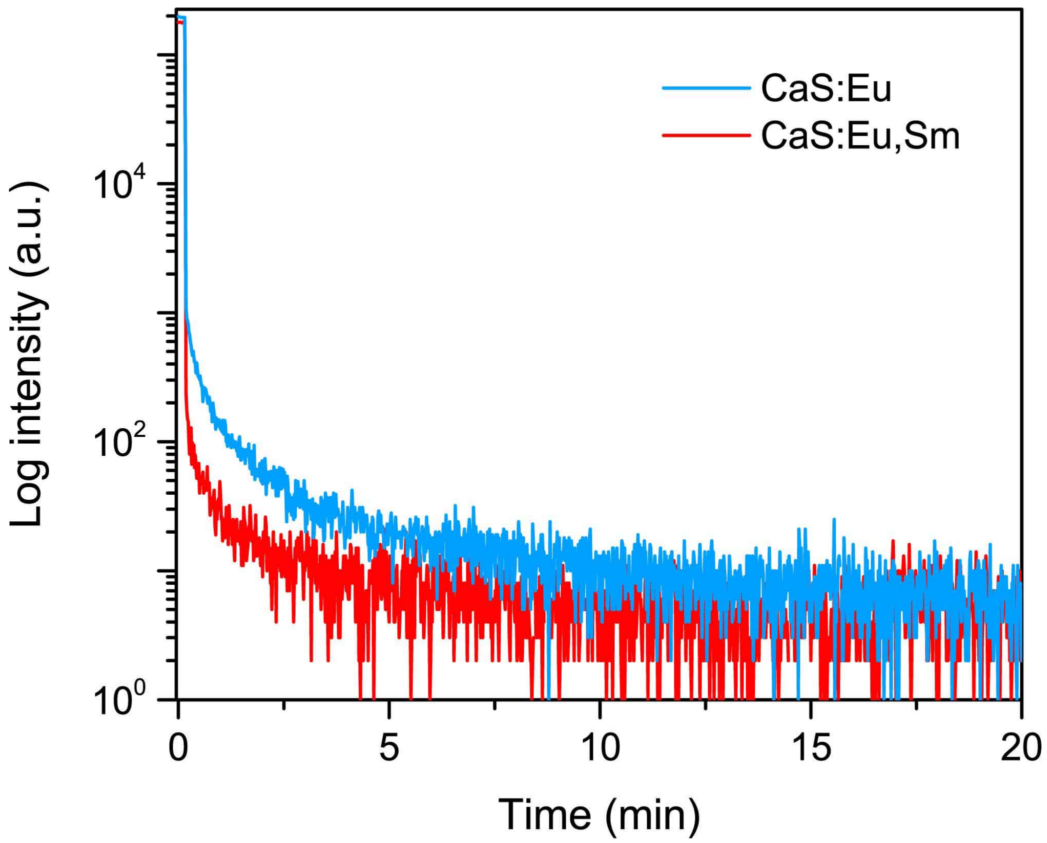

Figure S8. Persistent luminescence (PersL) decay curves of CaS:0.06%Eu^{2+} and CaS:0.06%Eu^{2+}, 0.006%Sm^{3+} NCs by monitoring the Eu^{2+} emission at 650 nm after the samples were illuminated with a household white LED (1 W) for 5 min, showing very weak PersL from the NCs with a duration about 10 min. Sm^{3+} co-doping was found to degrade the intensity and afterglow time of the PersL by introducing deep traps into the NCs.

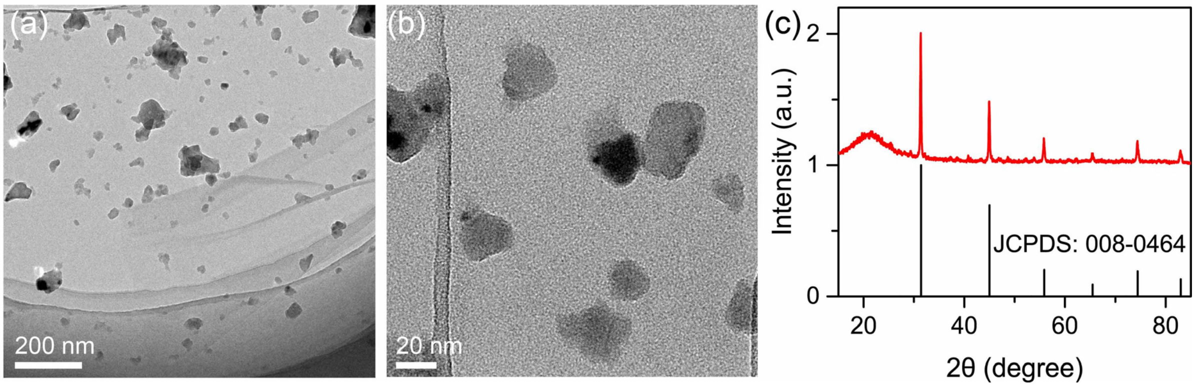

Figure S9. (a, b) TEM images (~0.2 mg mL^{-1}) and (c) XRD pattern of the annealed CaS:0.06%Eu^{2+}, 0.006%Sm^{3+} NCs. The annealed NCs preserved cubic phase of their pristine NCs. The mean size of the annealed NCs was calculated to be 34.6 ± 4.2 nm from the XRD pattern by using Debye-Scherrer equation.

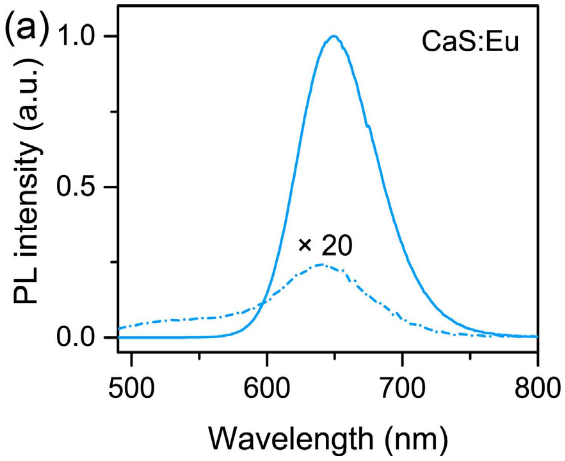

Figure S10. Comparison of the PL emission spectra of (a) CaS:0.06%Eu^{2+} and (b) CaS:0.06%Eu^{2+}, 0.006%Sm^{3+} NCs before (dash-dot line) and after (solid line) thermal annealing under excitation at 470 nm. After annealing, the PL intensities of CaS:Eu^{2+} and CaS:Eu^{2+},Sm^{3+} NCs were remarkably enhanced by factors of 101 and 43, respectively.

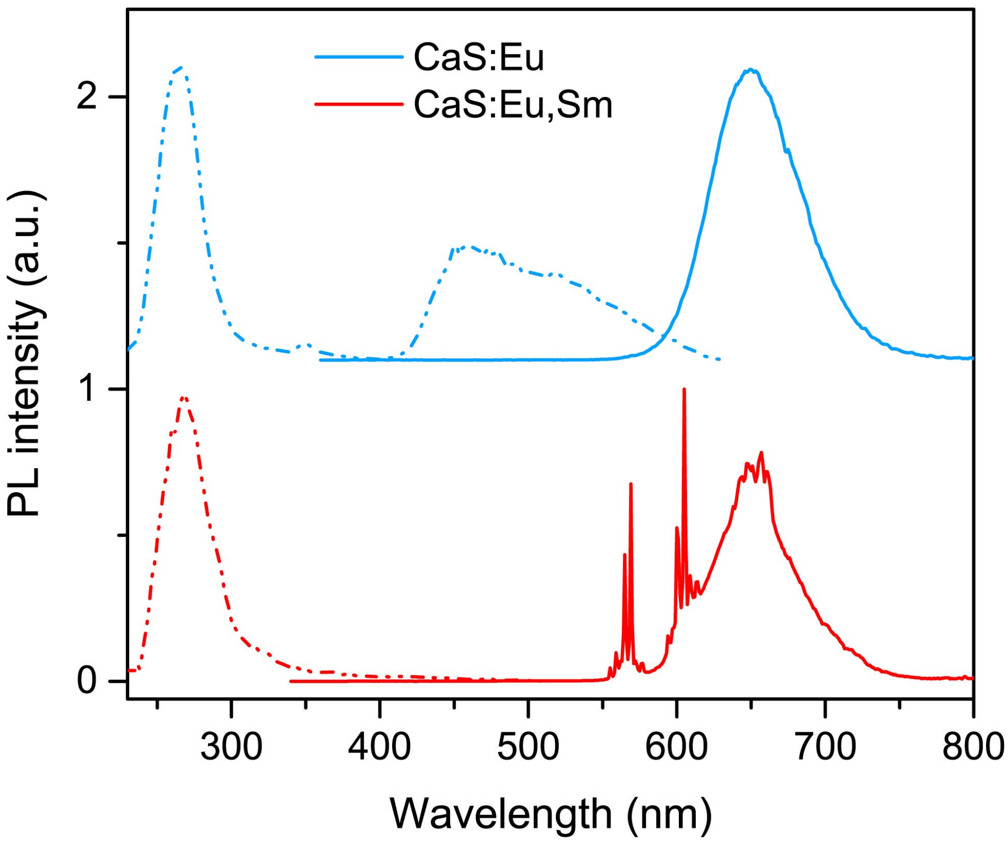

Figure S11. PL excitation (dash-dot line) and emission (solid line) spectra of the annealed CaS:0.06%Eu^{2+} (λ_{em} = 650 nm and λ_{ex} = 270 nm) and CaS:0.06%Eu^{2+}, 0.006%Sm^{3+} NCs (λ_{em} = 568 nm and λ_{ex} = 270 nm). In comparison with the pristine NCs, the defect-related excitation band at 325 nm and emission band at 400 nm disappeared in the annealed CaS:Eu^{2+} and CaS:Eu^{2+},Sm^{3+} NCs. This suggests that thermal annealing can effectively eliminate the detrimental defects in the lattice of the NCs induced by wet-chemical synthesis.

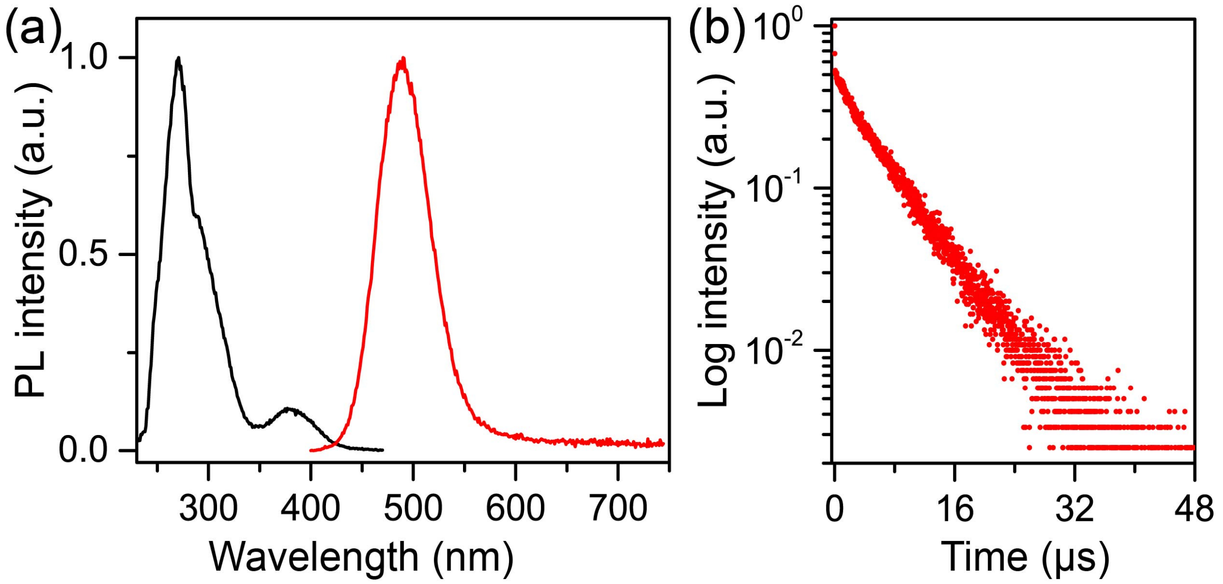

Figure S12. (a) PL excitation spectrum (black line, λ_{em} = 490 nm), PL emission spectrum (red line, λ_{ex} = 380 nm), and (b) PL decay of the annealed undoped CaS NCs. Upon excitation at 380 nm, a broad emission band at 490 nm was observed, which may be ascribed to the emission from new defects such as sulphur vacancies in CaS lattice formed during the thermal annealing process. By monitoring the defect emission at 490 nm, two excitation bands at 270 nm and 380 nm were detected, corresponding to the host and defect absorption of the NCs, respectively. By single-exponential fitting to the PL decay curve, the PL lifetime of the defect states was determined to be 2.9 μs. These results demonstrate unambiguously that thermal annealing can bring about new defects in the lattice of CaS NCs which may serve as electron traps for the persistent luminescence (PersL) of the NCs.

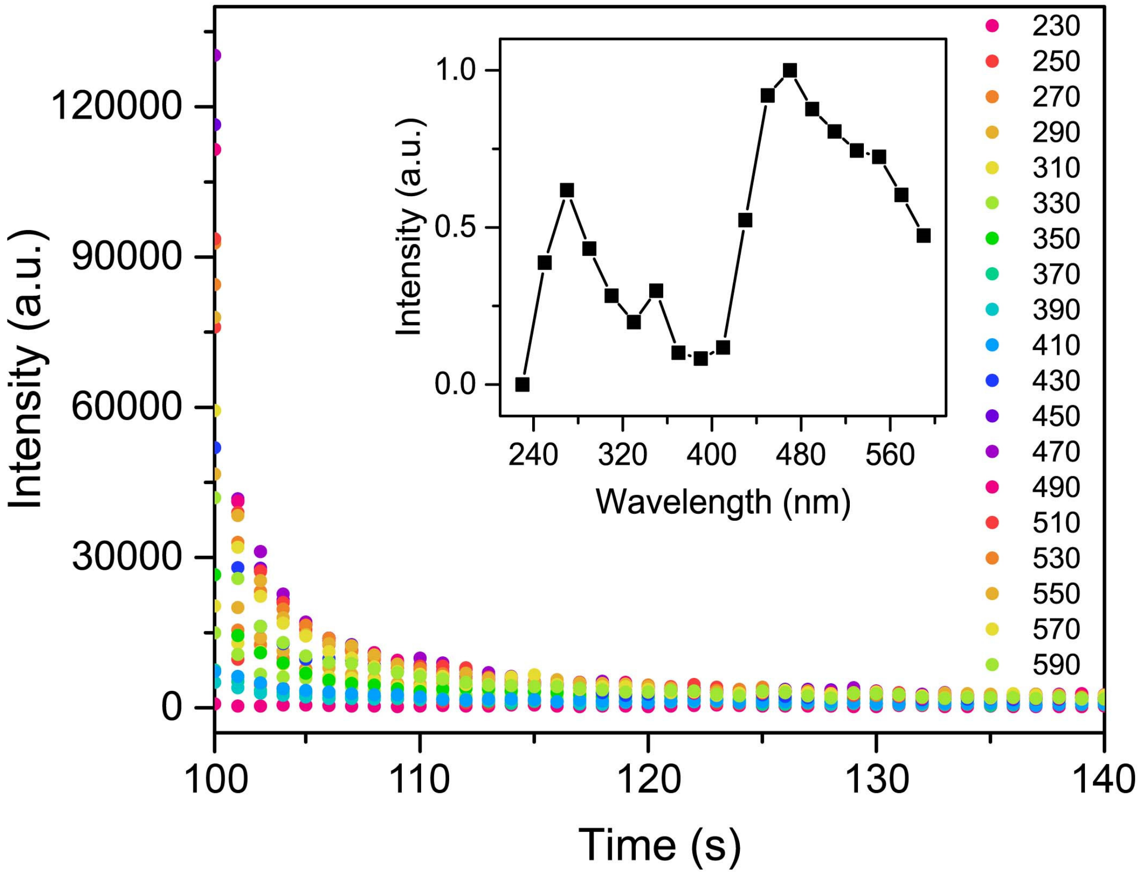

Figure S13. PersL decay curves of the annealed CaS:0.06%Eu^{2+}, 0.006%Sm^{3+} NCs with varying the excitation wavelength in the spectral region from 230 nm to 590 nm (λ_{em} = 650 nm). The inset shows the corresponding PersL excitation spectrum of the NCs obtained by integrating the PersL decay curves of the NCs at different excitation wavelengths.

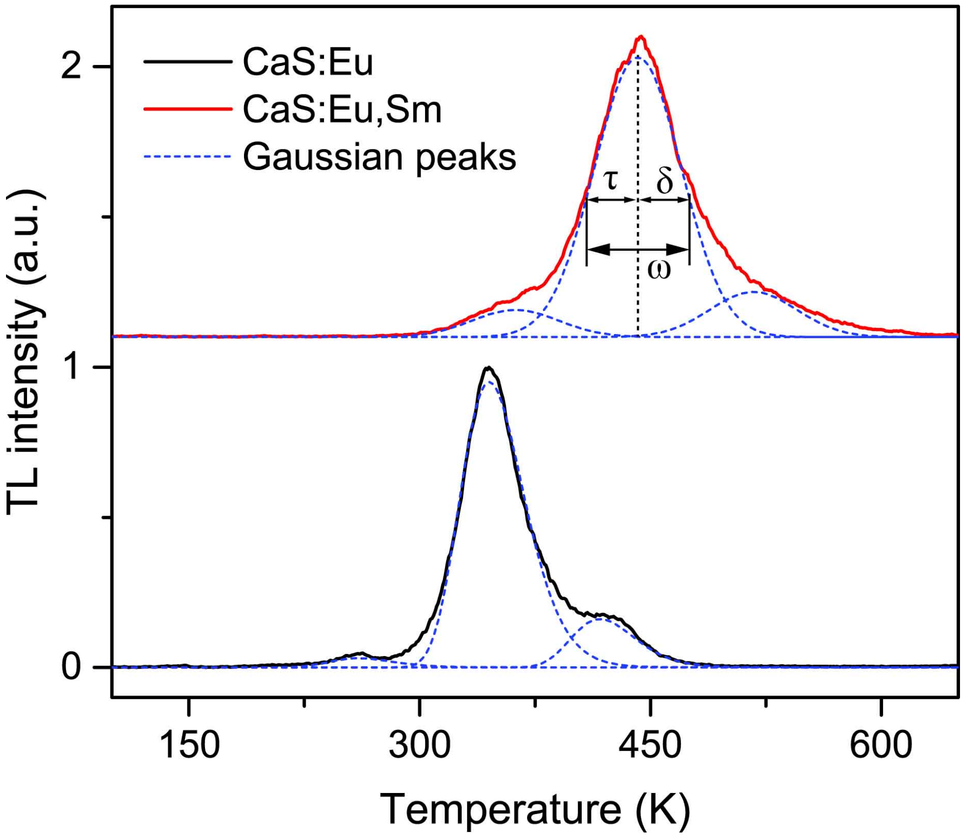

Figure S14. Thermoluminescence (TL) glow curves of the annealed CaS:0.06%Eu^{2+} and CaS:0.06%Eu^{2+}, 0.006%Sm^{3+} NCs. By Gaussian fitting to the TL glow curves, three TL glow peaks at 261, 344 and 416 K for CaS:Eu^{2+} NCs and three TL glow peaks at 361, 442 and 518 K for CaS:Eu^{2+},Sm^{3+} NCs were deconvoluted, corresponding to different trap depths in the lattice of the NCs. The trap depths of the NCs (E_t) were calculated by Chen's equation:

$$E_t = [2.52 + 10.2(\mu_g - 0.42)](\frac{k_B T_m^2}{\omega}) - 2k_B T_m,$$

where ω is the full width at half maximum (FWHM) of the TL band and defined as $\omega = \delta + \tau$, with δ being the high-temperature half-width and τ the low-temperature half width; the asymmetry parameter $\mu_g = \delta/\omega$; T_m is the temperature corresponding to the TL peak maximum. Based on Chen's equation, the trap depths were calculated to be 0.33, 0.63 and 0.92 eV for CaS:Eu^{2+} NCs and 0.47, 0.76 and 1.08 eV for CaS:Eu^{2+},Sm^{3+} NCs.

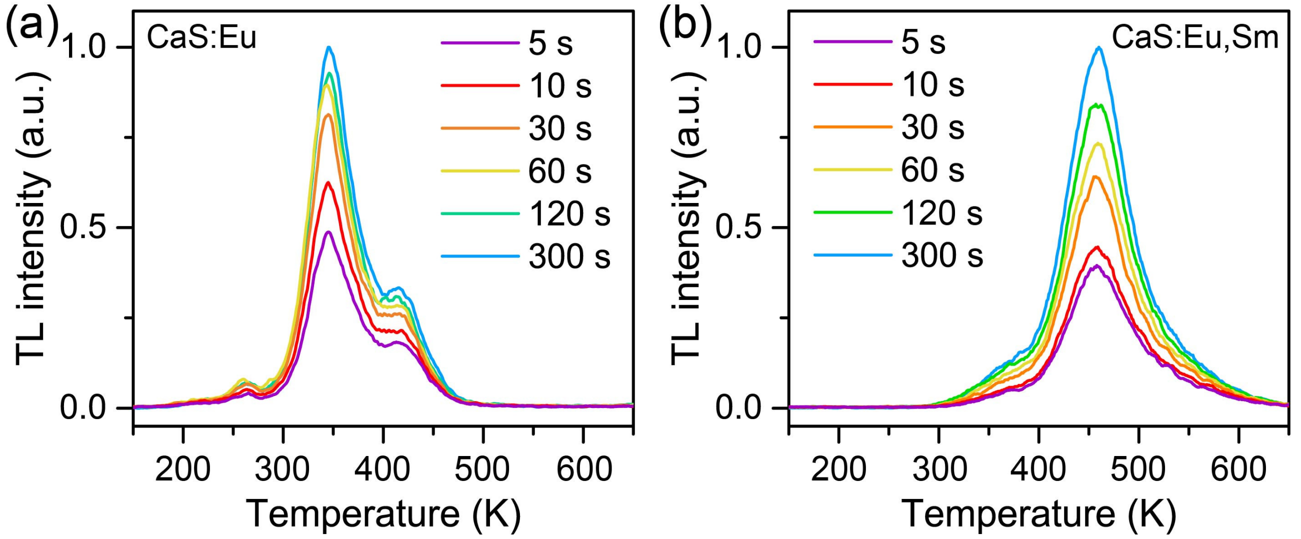

Figure S15. TL glow curves of the annealed (a) CaS:0.06%Eu^{2+} and (b) CaS:0.06%Eu^{2+}, 0.006%Sm^{3+} NCs with different pre-irradiation times. The samples were illuminated at 470 nm for different times at 300 K, and immediately after the cease of excitation, the temperature was allowed to fall to 100 K (30 s) and then rise to 650 K with a heating rate of 1 K s^{-1}. It was found that the pre-irradiation time had no noticeable influence on the TL glow peak positions of both CaS:Eu^{2+} and CaS:Eu^{2+},Sm^{3+} NCs, except for the increased TL intensity with increasing the pre-irradiation time as a result of increased number of trapped electrons.

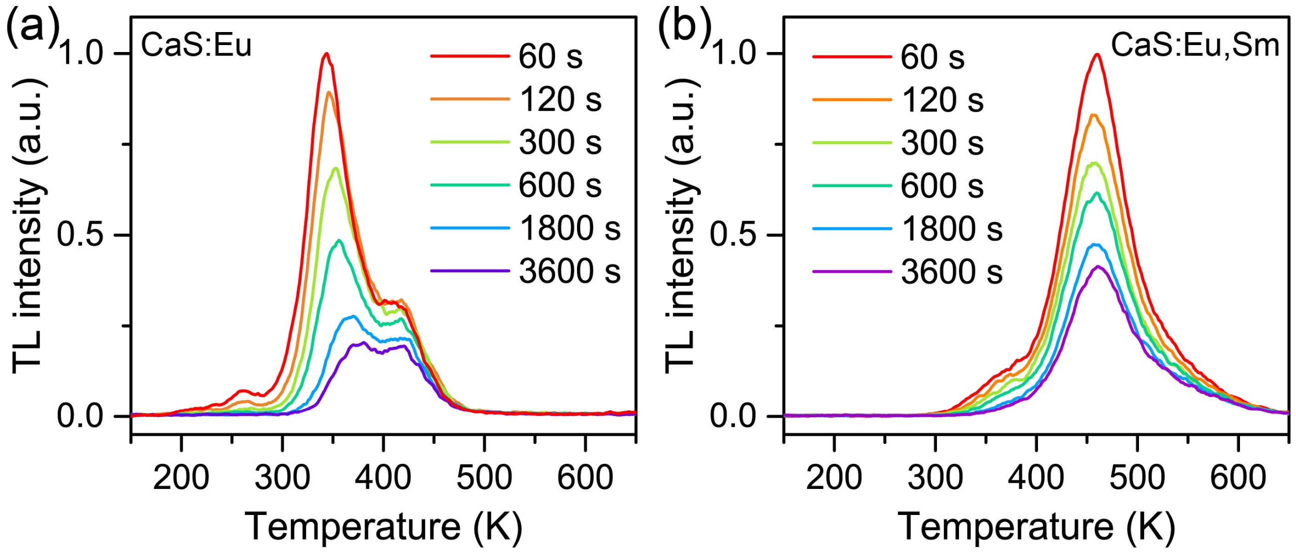

Figure S16. TL glow curves of the annealed (a) CaS:0.06%Eu^{2+} and (b) CaS:0.06%Eu^{2+}, 0.006%Sm^{3+} NCs with different delay times. The samples were illuminated at 470 nm for 5 min at 300 K, and after the cease of excitation, the temperature was kept at 300 K for different delay times and then allowed to fall to 100 K (30 s), followed by rising up to 650 K with a heating rate of 1 K s^{-1}. The TL glow peaks of CaS:Eu^{2+} NCs shift to higher temperatures with increasing the delay time, along with the decreased TL intensity, as a result of gradual release of electrons from the shallow traps of CaS:Eu^{2+} NCs. For CaS:Eu^{2+},Sm^{3+} NCs with deeper traps, the TL glow peak position shows only a slight shift to higher temperature because electrons in the deep traps could not be effectively released through thermal activation at room temperature.

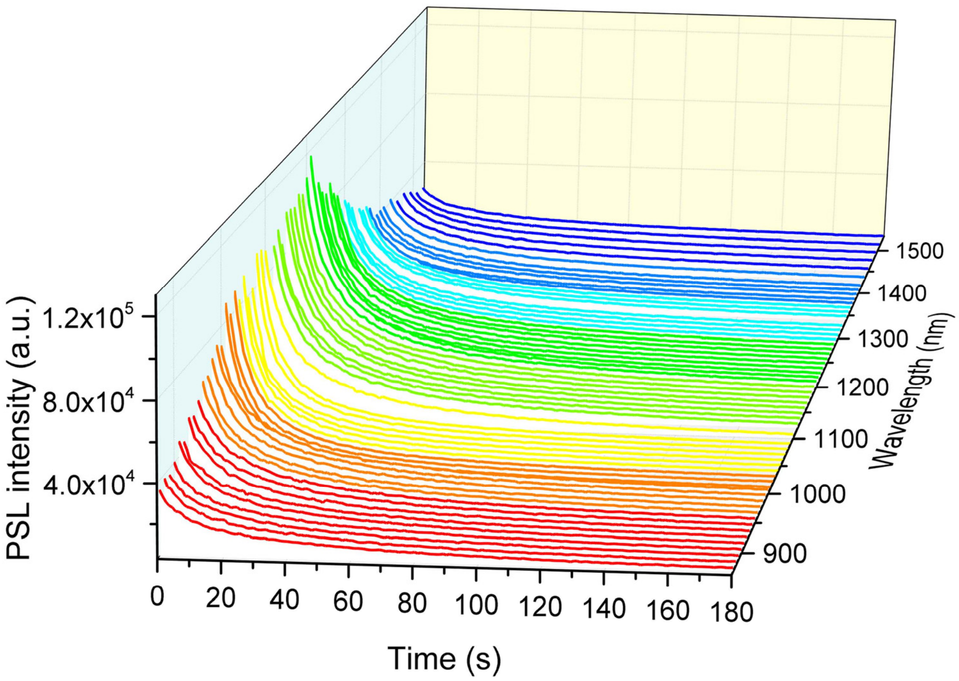

Figure S17. Photostimulated luminescence (PSL) decay curves of the annealed CaS:0.06%Eu^{2+}, 0.006%Sm^{3+} NCs by monitoring the Eu^{2+} emission at 650 nm under continuous excitation with a tunable OPO pulsed laser (410-2400 nm) in the wavelength region from 800 nm to 1600 nm. For each decay curve measurement, the sample was first charged with a white LED (1 W) for 5 min and the frequency and power density of the laser were kept constant. The PSL stimulating spectrum of the NCs was obtained by integrating the PSL decay curves at different stimulation wavelengths.

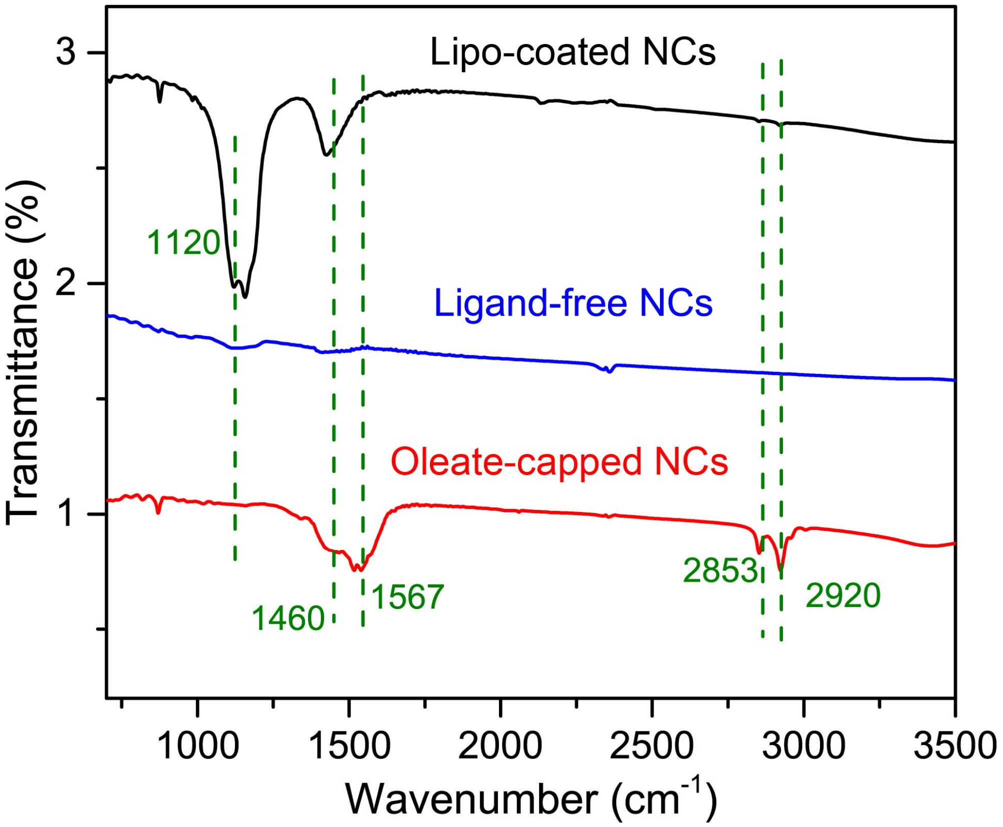

Figure S18. FTIR spectra of oleate-capped, ligand-free and Lipo-coated CaS:0.06%Eu^{2+}, 0.006%Sm^{3+} NCs. After thermal annealing, the original asymmetric and symmetric stretching vibrations of methylene (–CH_2–) in the long alkyl chain peaking at 2920 and 2853 cm^{-1}, and the asymmetric and symmetric stretching vibrations of carboxyl (–COO–) peaking at 1567 and 1460 cm^{-1} in oleate-capped NCs disappear in ligand-free NCs, indicating the successful removal of oleate ligands from the NC surface. The strong peak at 1120 cm^{-1} in Lipo-coated NCs is ascribed to the stretching vibrations of the C−O−C bond in DSPE-PEG-Biotin phospholipid, indicating that the DSPE-PEG-Biotin phospholipid was successfully coated on the NC surface.

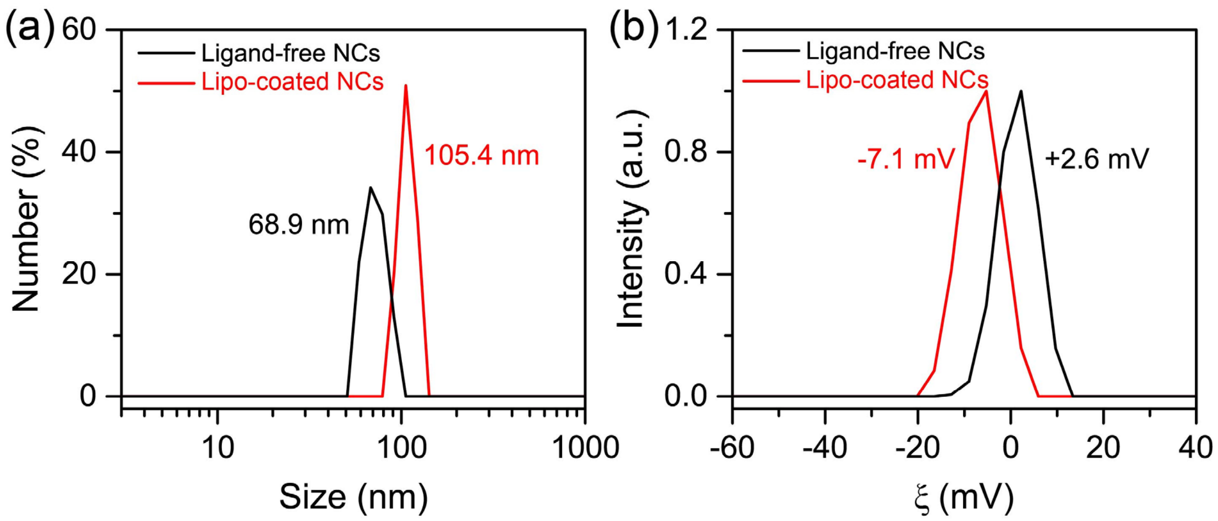

Figure S19. (a) Hydrodynamic diameter distributions and (b) ζ-potentials of ligand-free and Lipo-coated CaS:0.06%Eu^{2+}, 0.006%Sm^{3+} NCs dispersed in distilled water (pH 7.2) obtained from dynamic light scattering measurements (Nano ZS ZEN3600, Malvern). The increase in NC size and the change in ζ-potential of Lipo-coated NCs relative to ligand-free NCs confirm the successful coating of DSPE-PEG-Biotin phospholipid (Lipo) on the surface of the NCs.

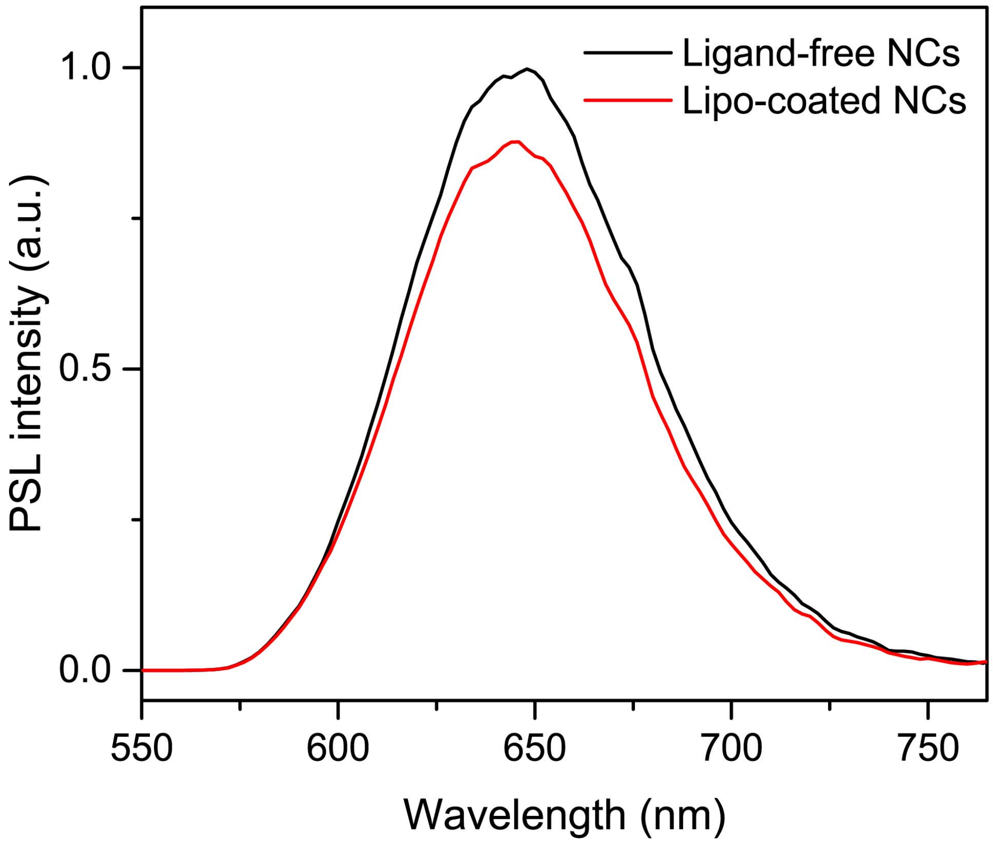

Figure S20. Comparison of the PSL emission spectra of ligand-free and Lipo-coated CaS:0.06%Eu^{2+}, 0.006%Sm^{3+} NCs under stimulation with a 980-nm diode laser at a power density of 1 W cm^{-2}, showing nearly identical PSL intensities of the NCs before and after Lipo coating.

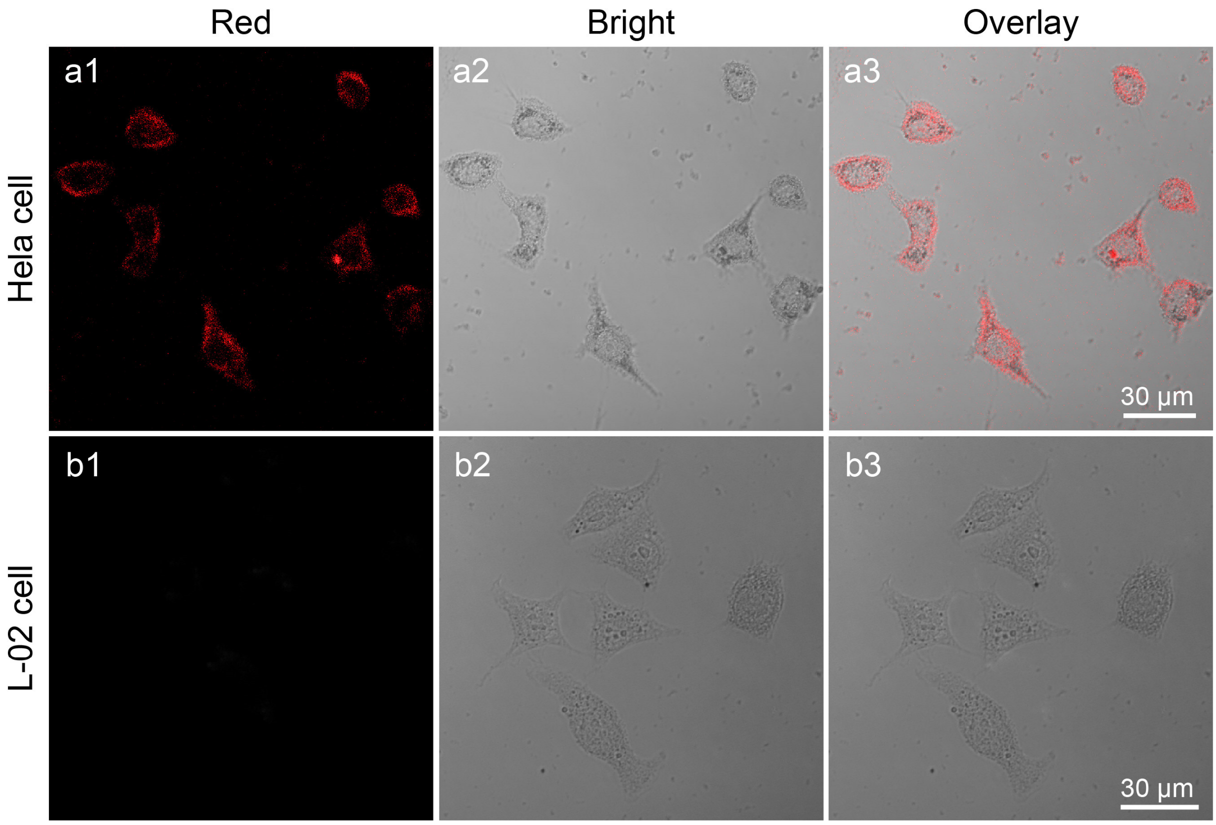

Figure S21. Confocal laser scanning microscopy images of (a1−a3) HeLa cells and (b1−b3) L-02 cells after incubation with Lipo-coated CaS:Eu^{2+},Sm^{3+} NCs (0.5 mg mL^{-1}) at 37 °C for 2 h. Intense red PSL of Eu^{2+} (λ_{em} = 640−680 nm, λ_{ex} = 1200 nm) was observed exclusively in HeLa cells after illumination with a white LED for 5 min. Panels 1 and 2 show the red PSL images and bright-field images, respectively. Panel 3 is the overlay images of panels 1 and 2.

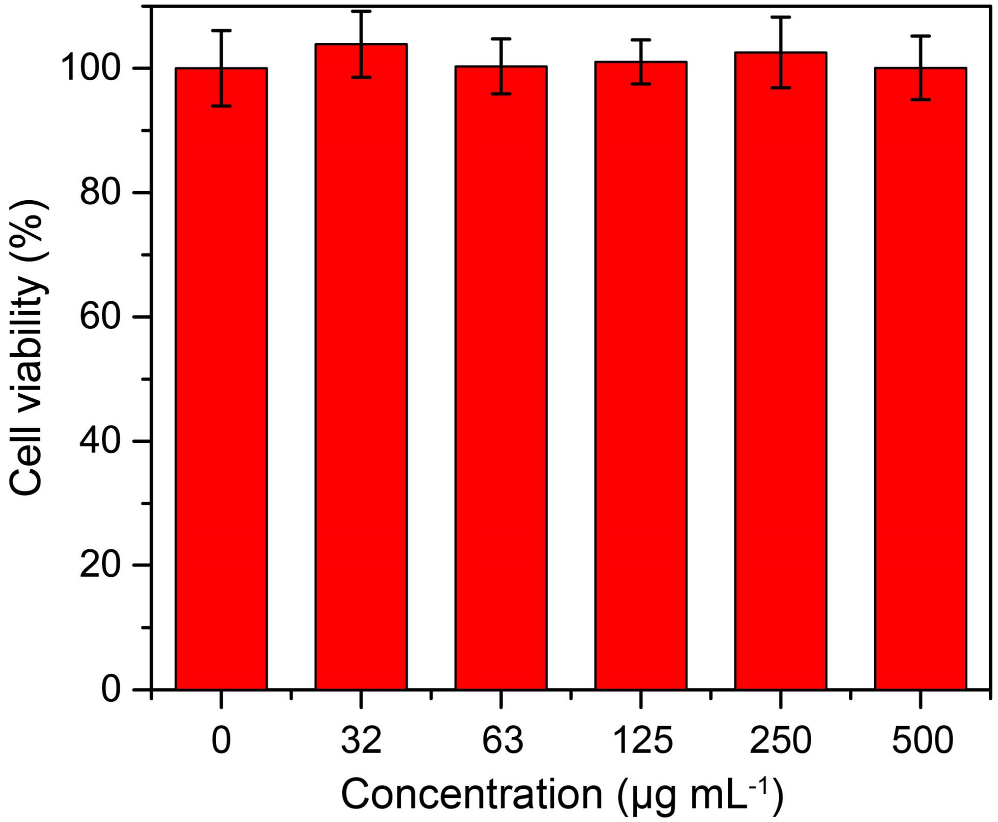

Figure S22. Cytotoxicity of Lipo-coated CaS:0.06%Eu^{2+}, 0.006%Sm^{3+} NCs against L-02 cells after co-incubation for 4 h by using MTT assay. The cell viability was determined to be larger than 98% even at a NC concentration as high as 500 μg mL^{-1}, suggesting that the Lipo-coated CaS:Eu^{2+},Sm^{3+} NCs are biocompatible and nontoxic to live cells.
